# Structural insights into fungal and human topoisomerase II with implications for in silico antifungal drug design

**DOI:** 10.1038/s41598-025-93122-1

**Published:** 2025-03-19

**Authors:** Subrahmanyam Sappati, Kavya Kondaka, Iwona Gabriel, Maciej Baginski

**Affiliations:** Department of Pharmaceutical Technology and Biochemistry, Gdansk University of Technology, Narutowicza St 11/12, 80-233 Gdansk, Poland

**Keywords:** K-loop, ScTopoII, hTopoIIα, TDL, Antifungal, Drug development, Computational models, Target identification

## Abstract

Topoisomerases are essential enzymes regulating DNA supercoiling and disentanglement, critical for genomic integrity. While topoisomerase inhibitors are well-established in anticancer and antibacterial chemotherapy, their potential as antifungal agents remains underexplored or even not proofed. This study investigates structural distinctions between *Saccharomyces cerevisiae* topoisomerase II (ScTopoII) and human topoisomerase IIα (hTopoIIα), aiming to identify if ScTopoII can be a selective target for antifungal drug development. A comprehensive sequence analysis, extending to various fungal strains and evolutionary ancient organisms, reveals dissimilarities in the transducer and transducer linker domains of these proteins, as well as in the lysine-rich K-loop region. Molecular dynamics simulations emphasize structural differences in the K-loop, α-helix (or helix-like region), and helix supporting loop region, as well as show unique patterns in hydrophilic and hydrophobic intramolecular interactions in ScTopoII. Moreover, phylogenetic comparisons support the importance of specific regions studied. The study includes topos from different organisms, highlighting discrepancies in helix stability near the K-loop and the role of helix supporting loop region. This broad analysis provides insights into the structural basis of human and fungal enzymes presenting potential pharmacophore “hot spots” in ScTopoII which may give hope for developing selective antifungal agents.

**Supplementary Information:**

The online version contains supplementary material available at 10.1038/s41598-025-93122-1.

## Introduction

**Fig. 1:**
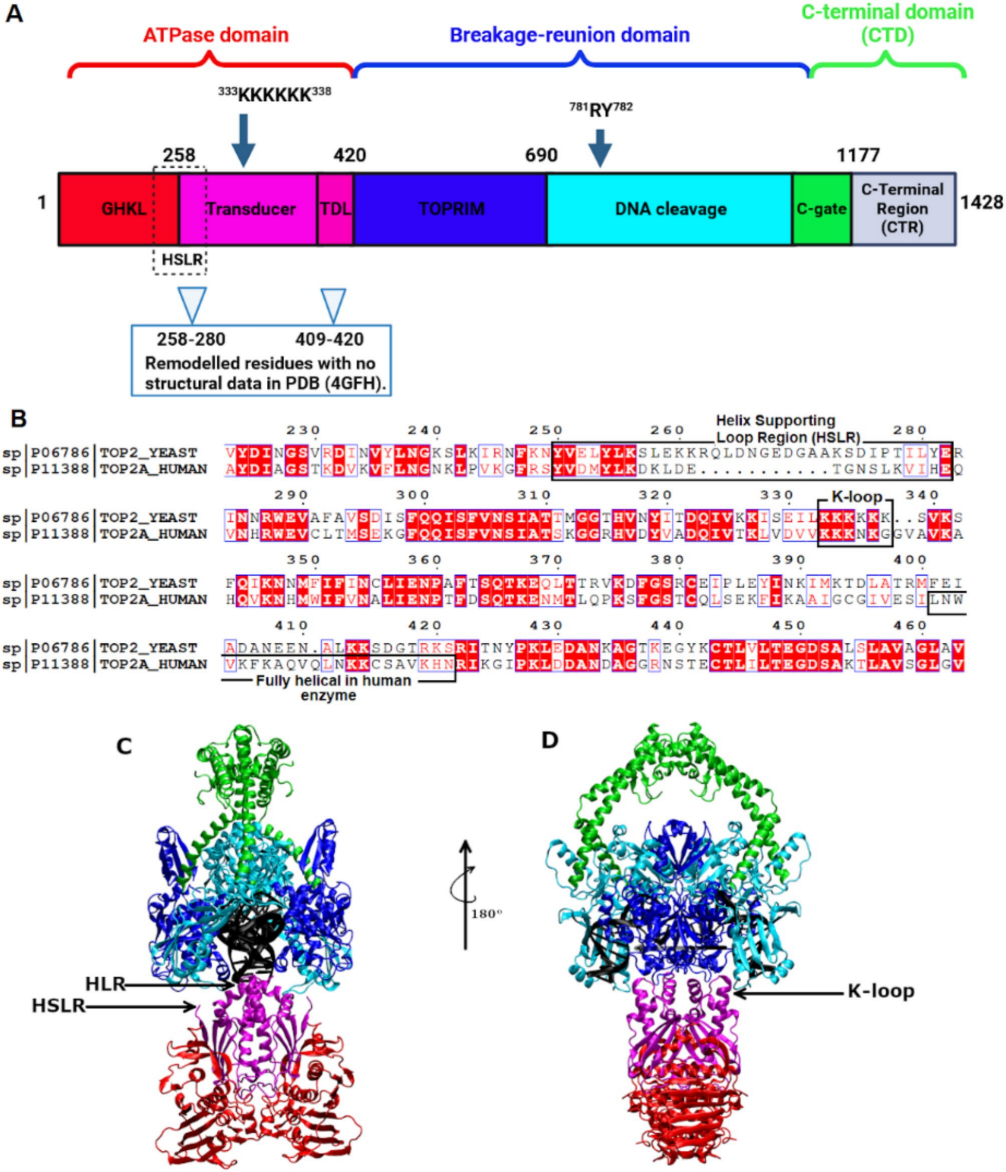
(**A**) Domain arrangement of *S. cerevisiae* topoisomerase II (ScTopoII). Transition state stabilizer (^781^R) and active site residue (^782^Y) as well as K-loop ^333^KKKKKK^338^ are indicated. Remodeled residues in ScTopoII structure are also presented. (**B**) Two sequence alignment focused on the transducer and TDL regions. Helix supporting region with additional amino acids for ScTopoII was also indicated (residues 250–282). The numbering of amino acid residues refers to ScTopoII. For hTopoII corresponding residues are 270–291. (**C**) A schematic representation of ScTopoII showcasing HLR, HSLR and (**D**) Rotated view of Figure C and showcasing location of K-loop. Further, we showcased our ScTopoII models in the SI Fig. S15.

Topoisomerases (topos) are essential enzymes that regulate DNA supercoiling and chromosome disentanglement, playing a pivotal role in maintaining genomic integrity and functionality in all eukaryotes^1^. These enzymes utilize a complex strand-passage mechanism to add or remove DNA supercoils and unlink DNA tangles or knots. Topos are classified as type I or type II depending on whether they catalyze the formation and re-ligation of single-stranded or double-stranded DNA breaks (DSBs), respectively^2^. Since topos are more often active in highly proliferative cells, human topos have become a target in the design and development of anticancer acting compounds^3,4^ Drugs classified as topoisomerase inhibitors can be divided into two groups: topoisomerase poisons or catalytic inhibitors^5^. The members of the first group (most of clinically used agents), such as etoposide, doxorubicin, and mitoxantrone, targeting topoisomerase II, lead to increases in the levels of TopoII: DNA non-cleavable covalent complexes. A second class of compounds, e.g. ICRF-187, inhibits TopoII catalytic activity, but do not generate high levels of TopoII covalent complexes^6^. Although those enzymes are significant molecular targets in anticancer chemotherapy very little is known if fungal topoisomerase II can be a useful and in particular selective target in antimicrobial chemotherapy. It is due to the fact that one may doubt whether there are sufficient molecular or functional differences between human and fungal enzyme to obtain selectivity in such antimicrobial approach. Some published data however, may support the idea that there are some structural differences between both topos as well as different sensitivity of both enzymes to already known human TopoII (hTopoII) inhibitors that can be explored^7–9^. It stems from the observation that activity of such compounds is different. On the other hand, looking for antifungal compounds with similar molecular mode of action, as those developed for human enzyme, carries the risk of high toxicity due to lack of selectivity. Thus, the aim of our work was to verify if there are important differences at molecular level within specific areas of the topoisomerase II structures (human and fungal) that have not yet been explored or even known. In a broader perspective such differences may define pharmacophore “hot spots” which can be further used in structure-based drug design process to develop new antimicrobial inhibitors. Such approach hopefully may led to find new topos inhibitors selectively acting against fungi. Recently a comprehensive crystal structure of a fully functional *S. cerevisiae* topoisomerase II (ScTopoII) homodimer complexed with DNA and a nonhydrolyzable ATP analog has been determined^10^. The different structures of human hTopoII are also available for comparison^11,12^ As structural domain organization of type II DNA topos is evolutionarily conserved, yeast enzyme also contains highly conserved functional motifs: the N-terminal ATPase domain, breakage-reunion domain and C-terminal domain (CTD) (Fig. 1A)^10–13^. Concerning the sequence of both enzymes (ScTopoII and hTopoII) it has been found that the major differences are in the ATP domain transducer region (Fig. 1B). These differences were major concern in our studies.

In particular, ScTopoII structure exhibits critical element, the K-loop, which is a lysine-rich loop present in the ATPase domain^10^. Yeast TopoII is unusual among eukaryotic type II topos in that it bears six lysines in its K-loop; most other orthologs possess only three or less of these residues. Moreover, the K-loop exhibits an unexpected interaction with the highly bent G segment of DNA and plays a vital role in both DNA-stimulated ATP hydrolysis and the overall activity of the topoisomerase. The exploration of the K-loop’s local environment holds immense importance, as it acts as a communication relay, connecting DNA binding to the stimulation of ATP turnover^10^. Additionally authors suggest that K-loop interactions with G-segment DNA seem to be crucial for eukaryotic TopoII enzymes due to a lack of this region observed in many prokaryotic type II topos^10^. It is also worth noting that molecular modeling studies of both enzymes were performed to investigate their catalytic functions, but these studies were conducted separately for each system rather than as a comparative analysis aimed at designing inhibitors for both enzymes^14–16^.

Therefore, comparative analysis was conducted by us on the transducer and transducer linker (TDL) domains (Fig. 1A) encompassing the K-loop of yeast ScTopoII and human topo IIα (hTopoIIα), utilizing full protein structures, deposited in PDB as 4GFH^10^ and 6ZY7/6ZY8^11^, respectively. The comparative sequence analysis revealed a prominent dissimilarity between the TDL domains of fungal and human TopoII (Fig. 1B), suggesting potential divergent structural functions or structural attributes in these two evolutionary lineages which can potentially be used in drug design process. In particular ScTopo II has in this fragment Helix Supported Loop Region, which is longer than in hTopoII (Fig. 1B). Taking into account available molecular structures of both yeast and human enzymes (4GFH and 6ZY7/6ZY8), respectively extensive molecular modeling studies were performed in current work. Overall, our comprehensive in silico investigation of the TDL domain of ScTopo II and hTopoII has uncovered intriguing similarities and differences in the interacting sites. In particular, unlike the human enzyme, one of the studied fragments in ScTopoII has a disordered 2D structure in the region (259–275) between GHKL domain and transducer region. Moreover, behaviour of helix supporting loop region is significantly different in ScTopoII compared to hTopoII. This finding reveals that all three regions presented in frames in Fig. 1B, nevertheless distant in sequence, are close in space and interplay with each other. To understand and explain these differences and fragments’ cooperation we performed phylogenetic analysis of TopoII sequences from different/several evolutionary (ancient) organisms^17,18^, which support our structural analysis. We analyzed sequences derived from organisms belonging to subgroups before (Amoeboza e.g. *Balamutia mandrillaris*) and after (Teretosporea e.g. *Sphaeroforma arctica*; Filasterea e.g. *Capsospora owczarzaki*) divergence to animals and fungi (Microsporidium e.g. *Rozella allomycis*) as it is stated in Fig. S1. Evolutionary divergence (Fig. S2) and sequence analysis (Fig. S1) suggest that all three regions had to evolve together. Altogether found differences give a hope that can be used in structure based drug design process to obtain selective agent acting as inhibitors only against fungal/yeast enzyme. In our future work we will undertake this challenge.

## Results and discussion

In order to challenge our hypothesis about possibility to use fungal TopoII as a target in antifungal chemotherapy we first performed sequence comparison of both enzymes ScTopoII and hTopoIIα (Fig. 1). As a result we selected the regions which potentially are important for enzyme function and are different in these two organisms (Table 1).

**Table 1:** Explored regions of sctopoii and hTopoII.

Key regions of TopoII	ScTopoII (Resname)	ResID	hTopoII (Resname)	ResID
K-loop	KKKKKK	333–338	KKKNKGGV	342–349
Helix like region (HLR)	TDLATRMFEIADANEENALKKDGTRKSRI	394–421	GIVESILNWVKFKAQVQLNKKCSAVKHNRI	406–435 (Helix)
Helix Supported Loop Region (HSLR)	VELYLKSLEKKRQLDNGEDGAAKSDIPTILYE	250–282	YVDMYLKDKLDETGNSLKVIHEQV	270–291
K-loop Supported Region (KSR)	—	—	GKILNVREASHK	488–499
DNA_ChainB	*CCTACTGCTAC*	1–11	CGCG*CATCGTCATCC*TC	1–17
DNA_ChainC	CGCG*GTAGCAGTAGG*	1–15	GA*GGATGACGATG*	1–13
DNA_ChainD	GGATGACGATT	1–11	CGCGCATCGTCATCCTC	1–17
DNA_ChainE	CGCGAATCGTCATCC	1–15	GAGGATGACGATG	1–13

- Comparative analysis of TDL domain in yeast and human topoII

To gain insight into such regions we focused on the local behaviour and function of TDL domain, encompassing the K-loop, helix like region (HLR), and helix supported loop region (HSLR) (See Table 1 for residue information). These fragments were the focal point of investigation due to its critical role in the functionality of the enzyme^10^. The comparative analysis revealed a prominent dissimilarity between the TDL of *Saccharomyces cerevisiae* and human TopoII, suggesting potential divergent molecular functions or structural attributes in these two evolutionary lineages. Regarding sequence conservation (ResID 250 to 450 of ScTopoII and ResID 270 to 464), the alignment analysis of ScTopoII and hTopoII exhibited a 42.9% identity, signifying some dissimilarities between the two species. Furthermore, the sequences shared a 63.0% similarity, encompassing not only identical residues but also those with comparable physicochemical properties. However, 7.4% of gaps were identified in the alignment, implying regions where amino acids were absent in one sequence relative to the other.

(b) Exploring structural differences through molecular dynamics simulations

In order to explore these structural differences and their potential functional role, molecular dynamics simulations were performed for both type of enzymes. We examined structures prepared for our study (described in Methods), namely ScTopoII and hTopoII based on PDB ID 4GFH for yeast as well as 6ZY7 and 6ZY8 for human representing conformational states 1 (closed) and 2 (pre-open), respectively. Utilizing the fully catalytic *Saccharomyces cerevisiae* topoisomerase II homodimer complexed with DNA at the DNA-gate, we conducted extensive microsecond-long classical molecular dynamic simulations with at least two replicas as described in methods. Our aim was to explore the overall dynamic behaviour of this molecular motor and investigate local conformational changes in the region of the key functional elements of transducer and TDL environment. Further, we focused on four important regions such as helix like region HLR-HLR interactions (in case of hTopoII helix-helix interactions), K-loop interaction with HLR, K-loop sensing DNA, HLR interacts with HSLR.

**Fig. 2:**
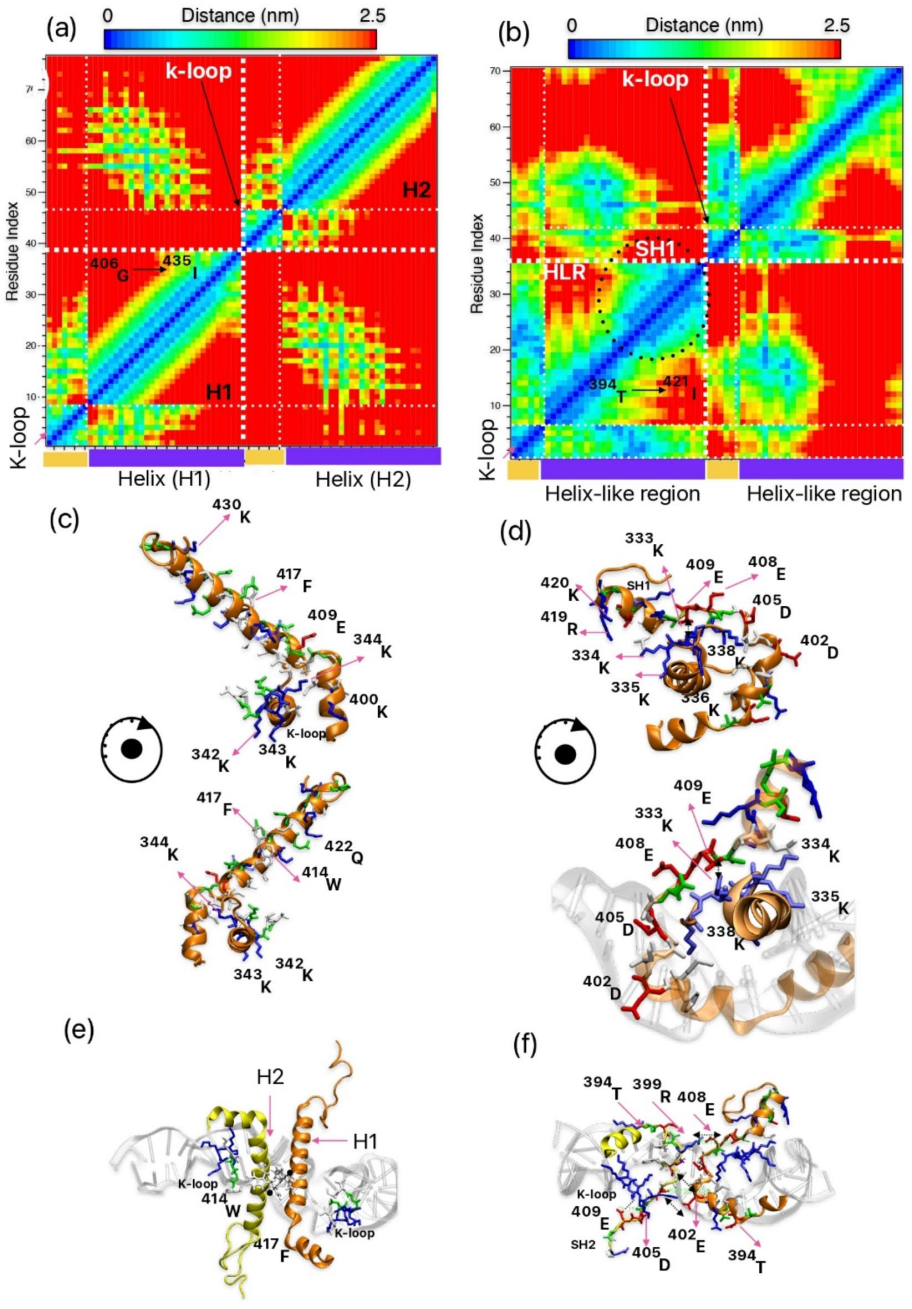
Distance matrices consisting of the mean smallest distance between residue pairs for (a) hTopoII (K-loop and α-helix) and (b) ScTopoII (K-loop and HLR) (See Table 1 for the sequence). Plots show short contacts between K-loop and helix region averaged over a time interval of 50 ns from 1 µs to 2 µs (short helix formation stabilized after 1µs) trajectory length. The short helix (SH1) formation from HLR region of ScTopoII encircled with black dashed lines. c) representative snapshot taken after 1 µs of hTopoII and showcasing interaction of K-loop and helix (showing just monomer), and rotated view is shown below e) same snapshot as c) is an interaction of inter chain α-helix (H1 and H2) (showing fragments from dimer). d) is a representative snapshot showcasing K-loop, HLR; taken after 1 µs of ScTopoII (showing just monomer) and rotated view is shown below. f) same snapshot as d) is an interaction of inter chain HLR regions of ScTopoII (showing fragments from dimer). Chain A (B) of the protein in light yellow (orange) and the colour coding of residues are based on residue type (non-polar residues (grey), basic residues (blue), acidic residues (red) and polar residues (green)).

To initiate our investigation, we prioritized the interchain interactions within the HLR-HLR of ScTopoII and between H1-H2 regions of hTopoII. Through extensive molecular dynamics (MD) simulations, we closely monitored the dynamic nature of HLR, in particular possibility to form stable secondary structures, and the short-range interactions between amino acids within this region. To elucidate these interactions, we generated distance contact maps (DCM) to visualize the spatial interactions between relevant amino acid pairs. The protein DCM represents the shortest distance of two given amino acids, averaged over a time interval of 50 ns from 1 µs to 2 µs. In Fig. 2a and b the regions colored blue to red show extent of interaction from maximum (shorter distance) to minimum (longer distance). This colour coding pattern is the same for all distance contact maps and these plots are diagonally symmetric. Analysis of DCM revealed close contact between the interchains (Chain A vs. Chain B) both in ScTopoII and hTopoII. Interestingly, we observed the α-helix (H1 and H2) to remain stable throughout the dynamics in hTopoII (Fig. 2c and e), while in ScTopoII, it exhibited a more dynamic nature and we did not observe any permanent stable secondary structure of the protein in our simulations (Fig. 2d and f). Notably, we observed for hTopoII a strong π-π stacking interactions between the tryptophan residue ^414^W and phenylalanine residue ^417^F, located in adjacent helices (H1 vs. H2) and the minimum distance of π-π stacking maintained around 3.4 Å (Fig. 2c and e). Further, we found supported interactions OH-π interactions between ^417^F-S^428^ (Fig. S3). In ScTopoII, interactions were mostly VdW or Coulombic interactions between ^403^I-E^402^, ^402^E-D^405^, and ^399^R-E^408^ (Fig. S4). For ScTopoII HLR appeared to be less organized with only short fragment forming helical structure (so called “short helix”) SH1 and SH2 within the second monomer of dimeric enzyme (encircled in Fig. 2b). Furthermore, we observed formation of SH1 after 1µs of production run (see Fig. S5 for the comparison of initial frame and representative snapshot after 1µs of one of the trajectory) till end of the simulation. This SH1 is part of HLR formed between ^409^N to K^420^ and further we found that SH1 in HLR is occupied with positively charged amino acids and remaining part of HLR spanning from ^395^D till ^410^N (closer contact of K-loop) in ScTopoII is rich in negatively charged amino acids (Table 2). In case of hTopoII, helix region features basic amino acids concentrated on one face of a helix, while the opposite side hosts non-polar aliphatic and aromatic groups, suggesting an amphipathic structure of the proteins. In contrast, fungal TopoII displays acidic residues in the HLR region that engage in intramolecular interactions and further interact with the K-loop, potentially stabilizing specific protein conformations and influencing enzyme regulation. Additionally, positively charged (basic) amino acids in fungal Topo II form a short helix.

**Table 2:** The number of positive or negative charged amino acids in the Helix (like) region (corresponding in yeast to ^394^T-K^420^ and in humans ^406^C-K^430^) as indicated in Fig. S2.

Source of TopoII	No. of positive/negative charged amino acids in K-loop	No. of positive/negative charged amino acids in Helix (like) region	No. of positive/negative charged amino acids closer to K/*R*-loop
*Saccharomyces cerevisiae*	6/0	4/6	1/5
*Homo sapiens*	4/0	5/1	2/1
*Rozella allomycis*	4/0	4/2	1/1
*Balamuthia mandrillaris* (R-loop instead of K-loop)	4/2	5/4	2/2
*Capsospora owczarzaki*	2/0	7/4	4/4
*Sphaeroforma arctica*	4/0	5/2	2/1
*Candida albicans*	5/1	4/5	1/3
*Candida glabrata*	6/0	5/4	0/4

To understand the dynamical behaviour of HLR in ScTopoII, we then shifted our focus to the K-loop (closer to HLR), a highly positively charged region known to play a significant role in the mechanism of Topo II. We monitored the dynamic interactions of the K-loop with the HLR in the case of ScTopoII (Fig. S6) and with H1 or H2 in the case of hTopoII (Fig. S7). Figure 2d depicts a representative snapshot in top view (rotated view is shown below) of K-loop of ScTopoII and K-loop is in licorice form (for clarity we are not showing hydrogen atoms); further we showcased first part of HLR (in licorice form) which is closer to ^337^K and ^338^K (K-loop). We observed strong hydrogen bond interactions between ^338^K and the region of HLR enriched in acidic (four negatively charged) residues (^402^D, ^405^D, ^408^E, ^409^E); further noting ^333^K-^401^F charge-π interactions during the dynamics. Such interactions are not present in case of hTopoII, which has just one negatively charged residue in this region. In consequence the residues from HLR in ScTopoII interact with K-loop and at the same time disturb formation of perfect helix. Thus, the presence of strong H-bond and electrostatic interactions appears to influence the structural arrangement of the HLR, hindering its ability to adopt a well-defined secondary structure, such as a perfect helix. (as shown in Fig. 2d). Further worth noting that AlphaFold3 predictions suggest that the HLR, as such, has the potential to form a helix. However, the observed interactions between the helices, as shown in SI Figure, create spatial constraints between the Transducer and TOPRIM domains in ScTopoII.

In case of human TopoII, we observed a contrast combination of amino acids in this region in comparison with ScTopoII, In case of K-loop interaction with helix, dominated VdW/Coulombic interactions (^344^K-V^408^, ^344^K-L^412^, ^345^N-S^410^) were observed (Fig. 2c). As discussed earlier, the helix part is dominated by positively charged amino acids, which then repel with the K-loop of hTopoII (Table 2). Such situation enables to keep stable α-helix in hTopoII (Fig. S8).

To understand the behaviour of helix, we remodeled HLR with Molecular Operating Environment (MOE) (as discussed in methods). Our findings revealed that the transition from the coil structure to the partial helix form destabilizes the helical structure, leading to a return to a disordered loop structure and partial formation of short helix (SH1) as discussed earlier. However, crucial interchain (Chain A vs. Chain B) interactions between HLR regions, as discussed earlier, were maintained throughout these transitions. Interestingly, in the case of the perfect helix structure formation which was preserved throughout this particular simulation, it was observed a loss of interchain interaction between HLR1 and HLR2.

**Fig. 3:**
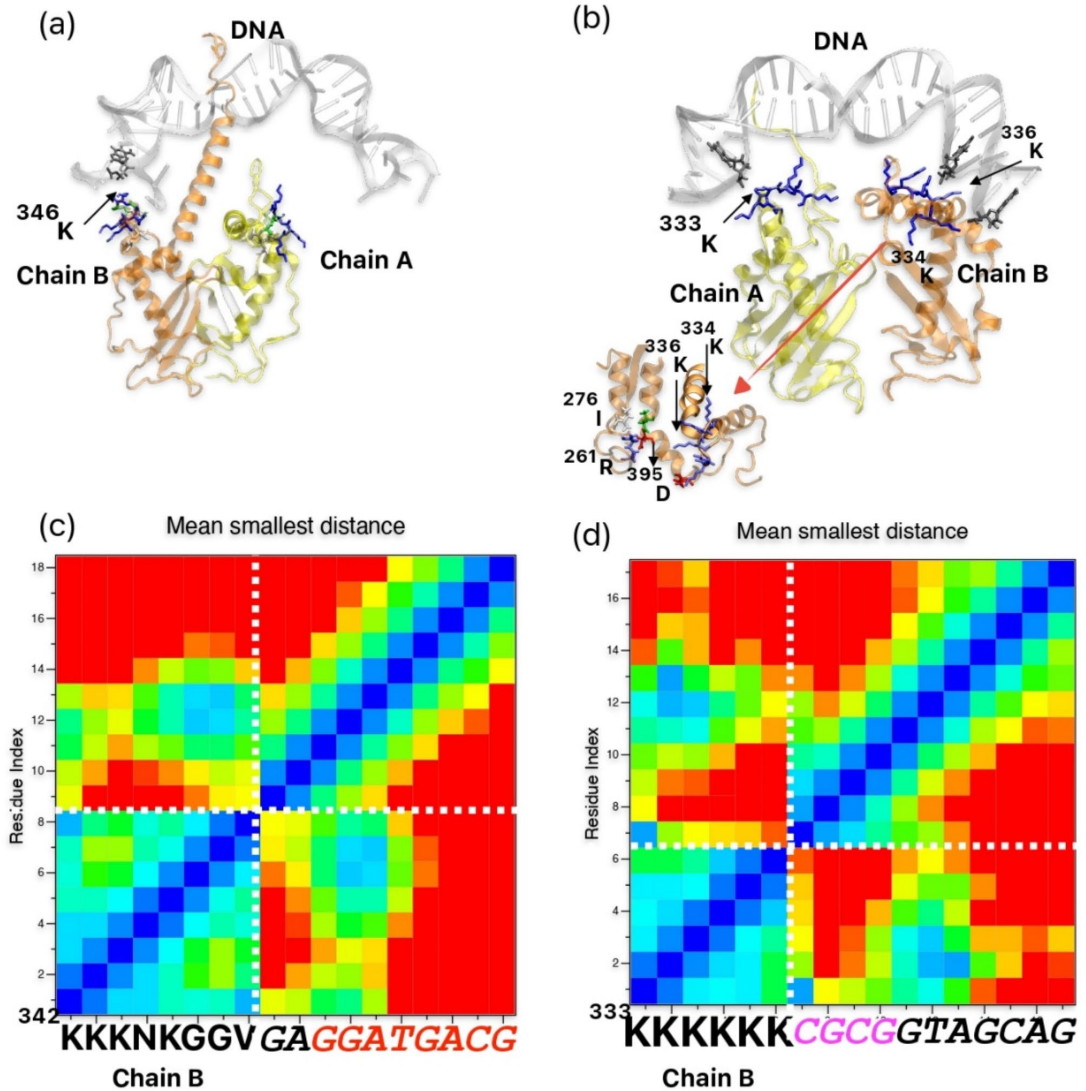
(**a**) and (**b**) represent snapshot showing interaction of K-loop with DNA in hTopoII and ScTopo II, respectively. (**c**) and (**d**) show distance map of K-loop sensing/interaction with DNA in hTopoII and ScTopo II, respectively. Here K-loop is in licorice with blue colour and closest nucleotides are in licorice, grey colour. Similarly, in case of hTopoII, K-loop is presented in blue licorice.

Concerning K-loop interaction with DNA, in case of hTopoII (chain B), there was initially no interaction between the K-loop and DNA. As the simulation progresses, K-loop (chain-B) of hTopoII sense the DNA while chain A drifts away from the DNA (as shown in Fig. 3a, Fig. S9-S10). We observed that ^346^K interacts with Guanine of DNA (Chain C) (See Table 1 for the sequence information of DNA) (Fig. 3c). In the case of ScTopoII, both chains A and B interact symmetrically with nucleic bases (Fig. 3b). In particular in ScTopoII, ^333^K-K^336^ sense the chain C of the DNA^4^GGTA^7^) (Fig. 3d).

In case of perfect helix as a starting point in the HLR region of ScTopoII, as mentioned earlier, we observed loss of interchain (Chain A and Chain B) of the protein interactions between HLR, and then lost symmetric sensing with DNA, the DNA domain inclined towards the K-loop of one of the chains. This deviation from symmetry resulted in minimized interactions between interchain HLR regions. Interestingly, this asymmetric interaction of DNA domain with K-loop is similar in hTopoII. These observations underscore the dynamic interplay between structural alterations in the HLR region and their impact on intermolecular interactions, shedding light on the intricate mechanisms governing the function of ScTopoII (Fig. S6-S7).

Other important regions of both proteins covered K-loop supporting regions. In case of hTopoII, we observed a K-loop supporting region (KSR: ^488^GKILNVREASHK^499^) interaction with important domains such as K-loop (^342^KKKNKGGVA^350^, H2, and DNA (Fig. S6); Specifically, ^498^HK^499^ of KSR are in close proximity to the K-loop, and maintain strong interaction throughout the simulation. Furthermore, we identified a helix-supported loop region (HSLR; ResID: 250–282) (as marked in Fig. 1; Table 1) in ScTopoII, which differs significantly from hTopoII (HSLR; ResID: 270–291), with a set of 11 amino acids absent in hTopoII. This HSLR of chain A interacts with the helix (H2) of chain B in hTopoII, whereas in the case of ScTopoII, the HSLR of chain A interacts with the HLR of the same chain A (Fig. 3b, Fig. S6). Interestingly, we found that HSLR region, in particular, residues ^268^E^269^D interact with major grove of DNA. This may indicate that the HSLR may play a key role in the rotational mechanism involved in the topoisomerase cycle as it interacts directly with DNA. This loop also interacts with another part of ScTopoII protein sequence ^921^S - ^932^D which strongly interacts with K-loop. In particular, K-loop interacts with ^924^D forming a strong salt bridge with an average distance of 2.5 Å. To understand the behaviour of HSLR region in ScTopoII, we remodeled it with MOE (as discussed in methods). All these forms prefer open (dynamical) conformation rather folded or other secondary structure such as beta sheet (See Fig. S11 for the comparison of initial and > 1µs conformation of HSLR region in ScTopoII). However, in all the equilibrated conformations it supports the HLR of ScTopoII (394 → 421).

For comparison, in case of hTopoII, we found an inter chain K-loop interaction with ^498^H which lasts throughout the simulations (Fig. S9). However, in case of hTopoII this region (HSLR) is small and formation of long loop which can reach DNA as it is in the case of ScTopoII is not possible. This shortening of HSLR in hTopoII occurred evolutionary since all organisms except yeasts and fungi have deletion in this region.

(c) Evolutionary Based Differences in Helix Like Region Stability and Helix Supporting Region:

To understand the discrepancy of helix or HLR proximate to the K-loop region between human and yeast enzymes evolutionary analysis was performed for different organisms. It is worth to note that, in the report by Dagmar Jirsova and Jeremy G. Wideman^18^, it is proposed that both the animal and fungal kingdoms share a common antecedent (clade) denoted as opisthokonta. However, their evolutionary trajectories have substantially diverged due to unique mechanisms. In the case of humans, this divergence is attributed to intricate processes such as gene duplications, gene fusion, and the advent of multicellularity, culminating in the intricate complexity observed in eukaryotic organisms. On the other hand, fungi have evolved via processes characterized by gene loss, gene fission, frequent horizontal gene transfers and specialized metabolic adaptations. These distinct evolutionary trajectories have, in turn, yielded distinct disparities in genetic constitution and functional attributes between humans and fungi^17^. This may also cover disparities in helix sequence following its distinct stability near the K-loop/R-loop environment in their respective enzymes. Therefore, we tried to look more precisely how these particular region looks like in different ancient organisms (Fig. S2). Based on the phylogenetic tree^18^, we considered 4 possible organisms such as *Balmuthia mandrillaris* (PDB ID: 7L6S), *Sphaeroforma arctica*, *Capsaspora owczarzaki*, and *Rozella allomycis* (Fig. S2). Along with these, we considered five more structures such as human TopoIIα (PDB ID: 6ZY7/6ZY8), human TopoIIβ, *S. cerevisiae* TopoII (PDB ID: 4GFH), and probable TopoII from *Candida albicans* (UniProt: P87078) and *Candida glabrata* (UniProt: O93794) (See Fig. S12-S13).

Initially, multialignment was conducted for all nine organisms, revealing significant disparities, particularly in the helix segment interacting with K-loop of TDL. To gain insight into the stability of the potential helix (HLR) and its interaction within the K-loop environment, specific regions were remodeled using AlphaFold, as detailed in Table S1 and Fig. S1^25^. These regions encompassed crucial K-loop and HLR, noting that X-ray and our MD studies indicated that this region of hTopoII forms perfectly structured helix. Initially, AlphaFold foreseen accurate helical structures of studied region for all nine organisms. To assess helix dynamical stability, extensive microsecond simulations (> 1 µs) were performed for eight organisms. Monitoring the average H-bond distances of n to *n* + 4 residues revealed distinct behaviour across organisms, compared with the HLR of ScTopoII and hTopoII (Fig. S14). In the AlphaFold geometry, the initial n to *n* + 4 H-bond distance was 0.35 nm. Notably, structures from fungal strains such as *S. cerevisiae*, *C. albicans*, and *C. glabrata* exhibited similar behaviour as ScTopoII and clear distinct behaviour compared to non-fungal organisms. In these cases monitored averaged H-bond length drifted in MD simulations above 0.4 nm (Fig. S14c). In contrast, hTopoII maintained a similar patterns concerning distance throughout the trajectory, similar as in structures from *Capsapora owczarzaki*, *Rozella allomycis*, and *Balamuthia mandrillaris*, oscillating around 0.35 nm (Fig. S14b). *Sphaereforma arctica* displayed behaviour similar to fungal strains, likely correlated with the number of negatively charged amino acids in the HLR (Refer to Table 2 and Fig. S12-S13 for DCM plots and representative snapshots of all 8 organisms).

Concerning HSLR similar alignment analysis was performed as for HLR (Fig. S1), and in this case it has been found that yeast and fungi are very distinct than other organism, including humans. The simulations performed in section b) indicate that longer loop in ScTopoII behaves in a different way compared to hTopoII. We can assume that sequences coming from different organisms having the same deletions as in human (Fig. S1) should behave in similar way. In particular, this fragment cannot sense DNA and similarly interact as in ScTopoII.

Taking into account sequence analysis of both enzymes as well as all studied regions (HLR or H, HSLR, K-loop) one may suppose that changes in all three regions of each enzyme are complementary in order to ensure functionality of enzyme. Concerning sequence, these regions are quite distant but taking into account their special location there are very close to each other.

(d) Structural hotspot in ScTopoII as potential binding regions for inhibitors

**Fig. 4:**
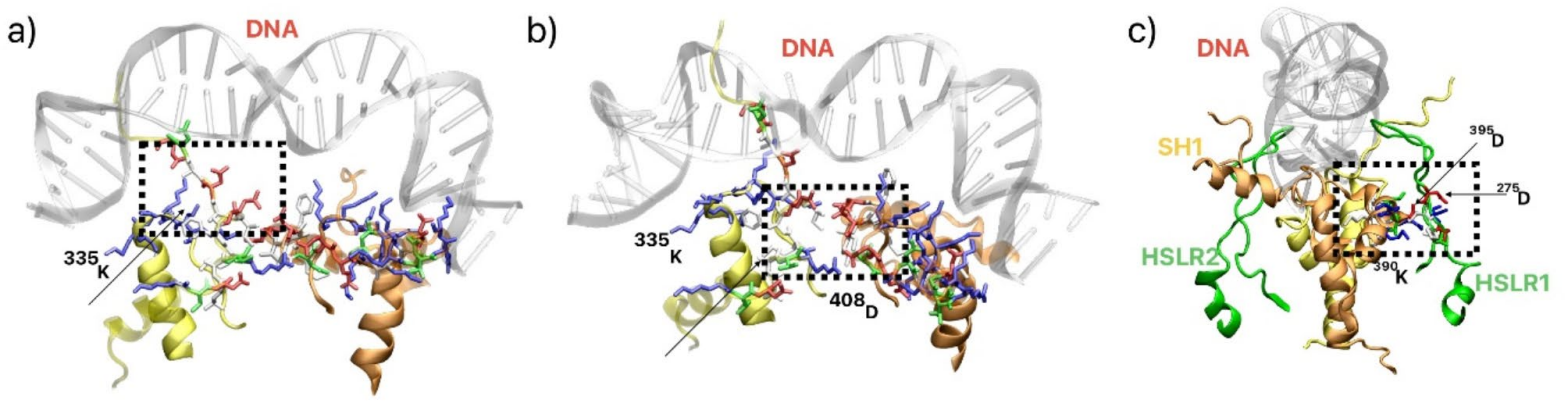
(**a**) Potential inhibitor binding areas (hotspots) in the middle region of K-loop and helix like region (HLR) of ScTopoII, (**b**) inter chain HLR-HLR and (**c**) interaction between helix supported loop region and helix like region.

Continuing our investigation, we delved into potential target areas (hotspots) for inhibitors within the transducer linker region. Our analysis revealed the formation of a cleft between the K-loop and HLR. Dynamic observations highlighted a strong interaction between the K-loop and HLR, as elaborated in the preceding section b). Notably, this region exhibits asymmetry in charge distribution, with one side of the cleft surrounded by positively charged residues and the other side enriched with D and E amino acids (Fig. 4a). The second identified potential binding area for inhibitors (hotspot resides) is located within the inter-chain region of the HLR, potentially serving as a hinge for inter-chain interactions within the HLR (Fig. 4b and Fig. S12). Further, we found a hotspot between helix supported region and helix regions and observed a salt bridge formation between ^399^R and ^275^D (Fig. 4c). It is worth mentioning that HSLR region is absent in the hTopoII as discussed earlier. Further, to understand the behaviour of HSLR, similar like HLR, we redesigned with MOE modeller and simulated extensively and found that loop is preferable (Fig. S13). Thus, these three regions (Fig. 4) are different in both type of enzymes and are closed to protein-DNA binding area which is responsible for topoisomerase II function.

Moreover, we employed DoGSiteScorer and MOE software to assess the ligandability of the identified hotspots in ScTopoII. For hTopoII, the analysis predominantly identified the DNA-binding and ATPase sites as the primary targets, with an average Simple Score of 0.4–0.6 across the top 15 ranked positions. ScTopoII displayed similar results for these canonical sites; however, two additional unique hotspots were identified in ScTopoII, each with a Drug Score exceeding 0.6 (Fig. S17): one located between the HLR-HLR regions and another between the K-loop and HLR. MOE results further corroborated these findings, highlighting the HLR-HLR region and the K-loop-HLR region among the top four ligandable sites (Table S3). These results emphasize the structural differences between ScTopoII and hTopoII, underscoring the potential of these unique hotspots for targeted drug development.

## Conclusions

Topoisomerase is an important enzyme for cellular function and therefore, in particular human one, has become a valuable target in anticancer chemotherapy. However, in recent years interest was also focused on fungal enzyme as potential target in antifungal therapy. In order to design selective inhibitors for fungal TopoII it is necessary to find differences in structure or function of these two enzymes. In our studies we undertook this effort and we have found substantial differences. The comparative molecular dynamics analysis of yeast and human topoisomerases performed in our studies has revealed intriguing distinctions in the structural and functional attributes of their TDL domains. The 3D structural differences of this domain stem from the sequence variations. This sequence divergence is observed as a consequence of evolution and it looks that fungi has unique different structure of this TDL domain. In particular, our sequence alignment study of different organisms indicate that fungi has a distant 1D structure of this TDL domain. Therefore, we focused on this domain as other parts (domains) of both enzymes have similar structure and consequently function. Our investigation focused on the dynamic behaviour of both enzymes emphasize structural differences in elements of this domain, namely the K-loop, α-helix (or helix-like region), and helix supporting loop regions, as well as show unique patterns in hydrophilic and hydrophobic intramolecular interactions in ScTopoII. The observed differences in helix formation and K-loop interactions between yeast and human topoisomerases provide valuable insights into their evolutionary trajectories. Moreover, found dissimilarities can potentially be regarded as a pharmacophore regions (hot spots) which can be used as molecular targets useful for designing selective inhibitors of ScTopoII. Especially interesting founding considers helix-like region and indicates that this region is not a rigid helix structure in fungal TopoII as it is in human one. Additionally unique structure found only in fungal TopoII of helix supporting loop regions which extends enough to interact with DNA can be also regarded as a potential target region. Our studies reveal that TDL domain behaviours differently in fungal than in human TopoII and this region is quite flexible, which is in agreement with very recent studies^16^. In summary identified regions of the chemotherapeutic applications (supported also by ligandability analysis) opens possibilities to design specific ligands to target these ‘hot spots’. Verification of our funding can be done only experimentally and we are on the way to design potential ligands. However, it takes time and therefore our data can be used also by other groups to verify our hypothesis.

## Methods

### Sequence alignment

We conducted an in-depth comparison of protein sequences for TopoII enzymes across various fungal strains: *Candida albicans* (Uniprot ID P87078), *Candida glabrata* (Uniprot ID O93794), and *Saccharomyces cerevisiae* (Uniprot ID P06786). Additionally, we explored these sequences in relation to human isoforms, Topo IIα (Uniprot ID P11388) and Topo IIβ (Uniprot ID Q02880). Expanding our scope to encompass evolutionary older organisms, we included representatives like *Sphaeroforma arctica* (A0A0L0FV23_9EUKA DNA JP610) from the *Teretosporea* lineage and *Capsospora owczarzaki* (A0A0D2 × 192_CAPO3, OX = 595528) from *Filastera*, as well as *Rozella allomycis* (A0A075AVT9_ ROZAC, OX = 988480) from *Microsporidium*, closely associated with fungal species. Our analysis extended to include representative from *Amoebozoa* (*Balamuthia mandrillaris*), utilizing PDB ID 7L6S for a comprehensive comparative study. We gathered Uniprot IDs and performed multi-alignment of FASTA sequences using the Clustal Omega tool within the Jalview software (Fig. S1)^19^.

### System preparation

Using the available X-ray crystal structure (PDB: 4GFH) as a reference, we built a full-length model of the molecular nanomachine ScTopoII. We addressed shorter missing loop regions in X-ray structure (residues 259–275, 409–420, and 603–606 in chain A, similarly same residues in chain B) through a loop refinement approach using Modeller software^20^. We selected the best 3D model based on the lowest possible energy criteria from the Modeller software. Our primary focus was on the K-loop and its surrounding regions. We eliminated the Mg^2+^ metal ions near the N-gate and removed AMP-PNP molecules from the crystal structure to understand the first step of catalytic cycle of topos. For MD simulations, we constructed two different structures of ScTopoII: in the first simulated system (i), we maintained the DNA in the uncleaved configuration as observed in the crystal structure, wherein both DNA chains of the G-segment within the topoisomerase IIA DNA-gate remained uncleaved, and the Tyr782 residues in both chains were restored to their native state. The second system (ii) was designated as apo version, where the DNA molecule was entirely removed.

In case of hTopoIIα we used two X-ray structures (PDB ID 6ZY7 and 6ZY8) and further to understand primary differences between fungal and human enzyme, we employed the charmm-gui builder^21,22^ to build just four missing residues (346–349; KGGV) in the K-loop region. The basic difference between (hTopoII) these two states is the following: PDB ID 6ZY8 is symmetrical with respect to all three major gates (N-, DNA- and C- gate) and PDB ID 6ZY7 is a bent conformation; as a result the K-loop come closer to DNA in this structure. In both cases, we studied our hTopoII systems with DNA. Moreover, to maintain consistency, in our approach we removed ligand EVP ((5 S,5aR,8aR,9R)-9-(4-hydroxy-3,5-dimethoxyphenyl)-8-oxo-5,5a,6,8,8a,9-hexahydrofuro[3’,4’:6,7]naphtho[2,3d] [1, 3]dioxol − 5-yl 4,6-O-[(1R)-ethylidene]-beta-D-glucopyranoside) and ANP (Phospho amino phosphonic acid-adenylate ester) molecules from the structures of the entire human Topo II nucleoprotein complex (PDB ID 6ZY7 and 6ZY8).

To understand the behaviour of the both HLR and HSLR region of ScTopoII, we employed molecular modeling techniques to redesign the HLR region within the protein from its native coil structure to a perfect helix using the MOE loop modeller and we designed three sets including the coil, partial helix, and full helix, with two copies of each. Subsequently, we conducted 1 µs simulations for each system. Overall we performed 8 µs simulations ScTopoII^23^.

To delve into the intricacies of the α-helix structures present within the TDL region we also studied separated helical fragments of TDL coming from different organisms. In particular, our approach involved selecting PDB entries and FASTA sequences for topos coming from different organisms present in Fig. S1. Appropriate 3D models of these regions were built. Nevertheless, our comparison centered on yeast and human topoisomerase II. The specific focus was on highlighted segments within the multiple sequence alignment shown in the Fig. S1, encompassing the K-loop and TDL region.

We employed the ColabFold v1.5.5 version to reconstruct eight structures coming from organisms (excluding hTopoIIβ due to its 98% similarity with hTopoIIα) present in the phylogenic tree (Fig. S1)^24^. This version of software integrates AlphaFold2 and RoseTTAFold, along with a fast multiple sequence alignment process facilitated by MMseqs2^24,25^ As highlighted in the Fig. S1 with parenthesis, which encompasses both K-loop and helix-like regions or perfect helix, we considered this sequence for analysis. Subsequently, we proceeded with classical MD simulations with the following protocol.

### Simulation details

The simulation protocol involved classical all-atom molecular dynamics (MD) simulations, which were carried out using GROMACS 2020 software^21,26^. The CHARMM36m force field was utilized in our study for all systems. The TIP3P model was employed for water molecules^21,22^ Original charge for all ionisable amino acids residues was preserved. Moreover, for all studied structures hydrogen atoms, lacking in X-ray were added in an automatic way. K^+^ and Cl^-^ ions were added into the solvent to reach a physiological salt concentration of 0.15 M and neutralize the system. The simulations were carried out in the isothermal-isobaric (NPT) ensemble using periodic boundary conditions in 3D cubic model with a minimum distance of 1 nm between the solute and the box edges (Table S1 and S2). The constant temperature was kept at 310 K using the v-rescale thermostat^27^ and the pressure was maintained at 1 bar semi-isotropically using Parrinello-Rahman algorithm^28^. Long-range electrostatic interactions were evaluated using the Particle Mesh Ewald (PME) method with a real-space cut-off of 1.2 nm^29,30^ Van der Waals interactions were evaluated using a smooth cut-off of 1.2 nm with a switching distance of 1 nm. Bond lengths for hydrogen atoms were constrained using the SHAKE (for water molecules) or P-LINCS algorithm (for all other atoms)^31,32^ The equations of motion were integrated using the leapfrog Verlet al.gorithm with a time step of 2 fs. Prior to the production simulations, each system underwent energy minimization and equilibration through short conventional all-atom MD simulations. There were two phases of equilibration. During the first phase of equilibration (20ns), position restraints were applied to the backbone atoms of the protein. In the second phase (100 ns) all restrains for backbone atoms were removed. Two random frames from the second phase of this restraint-free run were picked as initial coordinates for two independent unbiased MD simulations of ScTopoII and hTopoII, each lasting 1.5 µs. In total, we produced eight MD trajectories: two for ScTopoII apo version, two for ScTopoII with DNA, two for hTopoII (corresponding to the conformation from 6ZY7) and two for hTopoII (corresponding to the conformation from 6ZY8).

### Ligandability analysis

To perform this analysis we used on-line web server DoGSiteScorer^33^ and MOE software (active site finder subroutine). For this purpose we used pdb files of starting (X-ray) structures of studied systems and final ones coming from MD simulations.

## Electronic supplementary material

Supplementary Material 1

## Data Availability

Data generated or analyzed in this study are included in this published article and supplementary material files. Raw data supporting our results are available from authors S. S. (subsappa@pg.edu.pl), M. B. (chemmbag@pg.edu.pl) and I. G. (iwogabri@pg.edu.pl) on request.
