# Supplementary material for: Structural insights into fungal and human topoisomerase II with implications for in silico antifungal drug design
Source: Sci Rep. 2025 Mar 19;15:9467. doi: 10.1038/s41598-025-93122-1 (PMC11923201; doi:10.1038/s41598-025-93122-1)
Supplement: Supplementary file 1 — Supplementary Material 1 [file 41598_2025_93122_MOESM1_ESM.pdf]

Structural insights into fungal and human topoisomerase II with implications for in silico antifungal drug design

Subrahmanyam Sappati[$], Kavya Kondaka[$], Iwona Gabriel[$]*, and Maciej Baginski[$]*

[$]Department of Pharmaceutical Technology and Biochemistry, Gdansk University of Technology, Narutowicza St 11/12, 80-233 Gdansk, Poland.

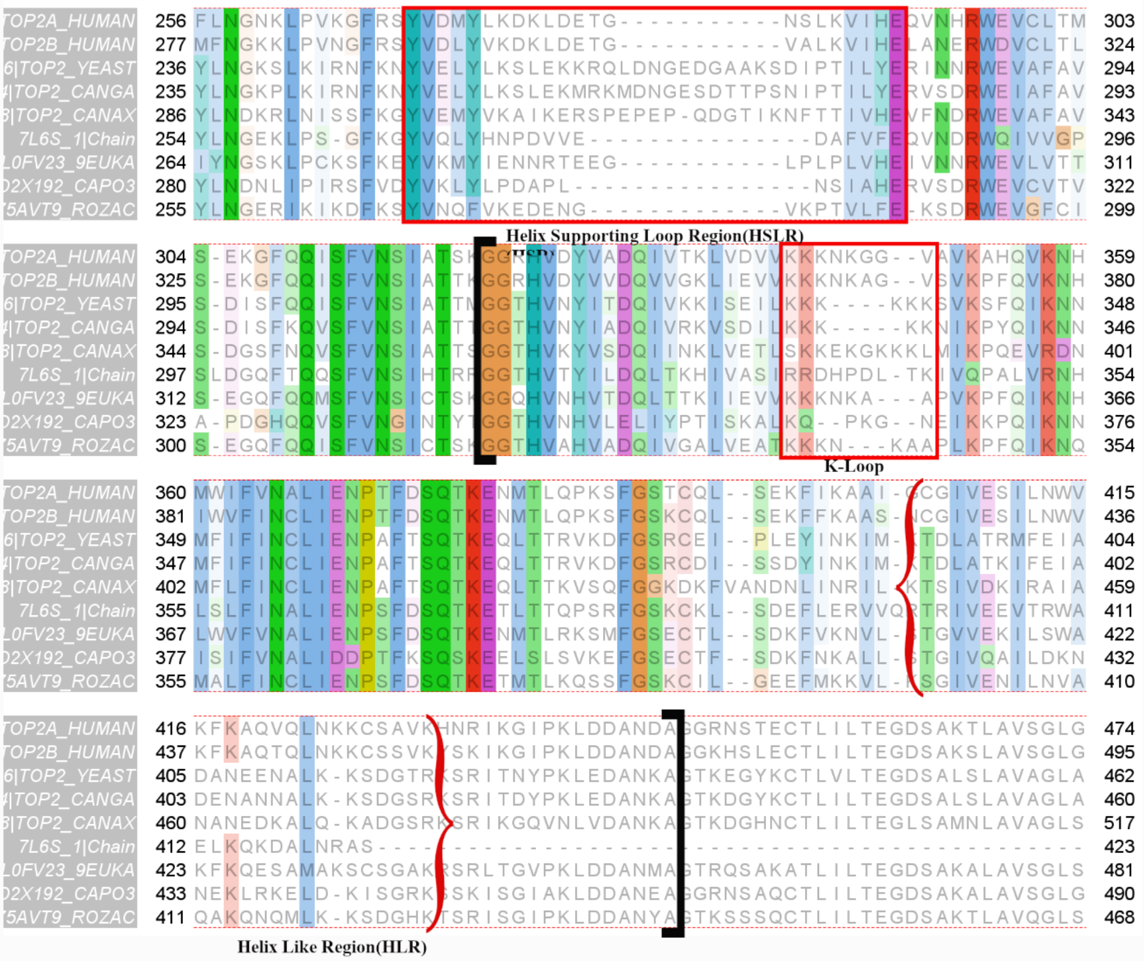

Figure S1: Multi-alignment of nine topoisomerase II sequences from different organisms, namely human TopoII isoforms (TOP2A Human, TOP2B Human), enzyme from *Saccharomyces cerevisiae* (TOP2 YEAST), *Candida albicans* (TOP2_CANAX), *Candida glabrata* (TOP2_CANGA), *Balamutia mandrillaris* (PDB: 7L6S), *Sphaeroforma arctica* (A0A0L0FV23_9EUKA), *Rozella allomycis* (A0A075AVT9_ ROZAC), *Capsaspora owczarzaki* (A0A0D2X192_CAPO3).

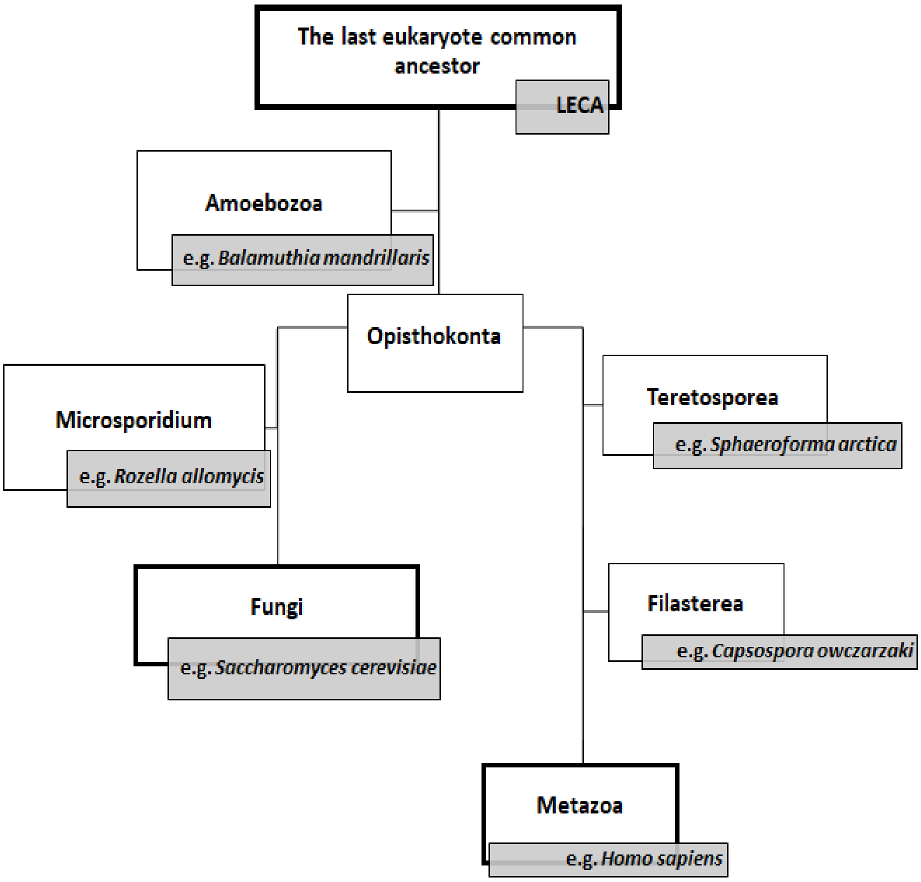

Figure S2: Major evolutionary trajectories of animals and fungi.

Table S1: Description of the simulations of the entire protein with and without DNA

Description of the System	Box_x (Å)	No. of atoms
ScTopoII + DNA (PDB ID: 4GFH)	212.288	669104
ScTopoII	207.769	632052
hTopoII+DNA (PDB ID: 6ZY7)	230.000	667615
hTopoII+DNA (PDB ID: 6ZY8)	204.299	861666

Table S2: Description of the simulations of the protein fragments. Topoisomerases sequences are derived from Uniprot or PDB: for *Saccharomyces cerevisiae* Uniprot ID P06786, human enzyme Uniprot ID P11388, *Rozella allomycis* A0A075AVT9|A0A075AVT9_ ROZAC, OX=988480, *Balamuthia mandrillaris* PDB ID: 7L6S, *Capsospora owczarzaki* A0A0D2X192|A0A0D2X192_CAPO3, OX=595528, *Sphaeroforma arctica* A0A0L0FV23|A0A0L0FV23_9EUKA DNA JP610, *Candida albicans* Uniprot ID P87078, *Candida glabrata* Uniprot ID O93794.

Description of the system	Box_x (Å)	No. of atoms
ScTopoII *(313G-425K)*	10.245	108650
hTopoII *(322G-437D)*	11.926	170647
Rozella allomycis (318G-431Y)	10.853	128500
Balamuthia mandrillaris (316G-424S)	7.8998	49661
Capsospora owczarzaki (341G-453E)	10.256	170647
Sphaeroforma arctica (330G-M444)	10.574	161607
Candida albicans (312G-423K)	11.461	151689
Candida glabrata (362G-423K)	11.356	181670

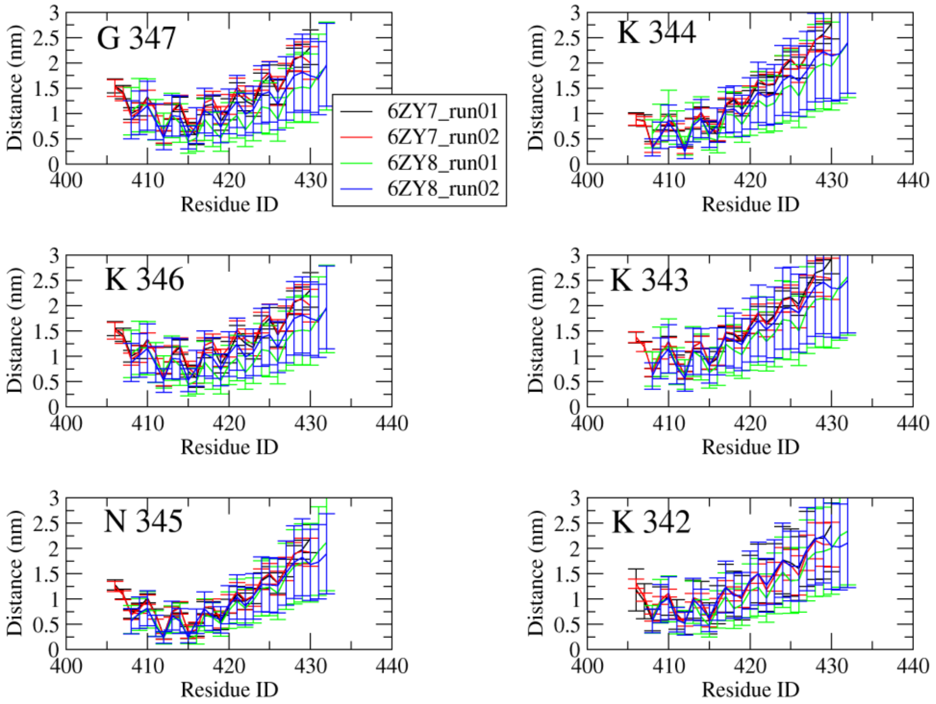

Figure S3: Average pair distance between K-loop (342K to G347) and Helix (H1) of hTopoII for 2 runs each state [closed and pre open] as described in the methods.

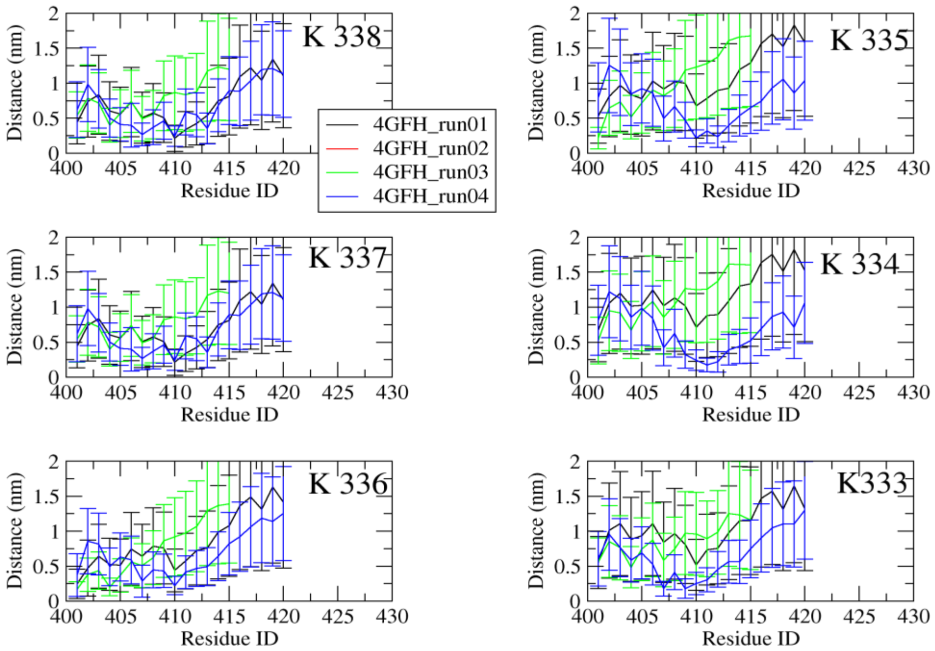

Figure S4: Average pair distance between K-loop (333K-K338) and HLR of ScTopoII for 2 runs as described in the methods (and 2 more copies for comparison).

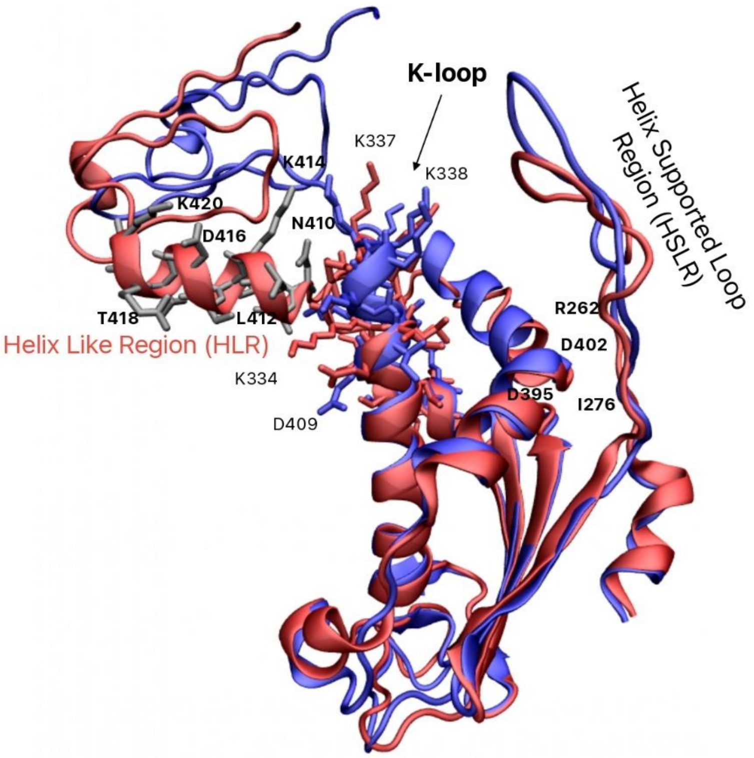

Figure S5: Comparison of initial and after 1 μs frames of ScTopoII (residues 250 to 450) (a) Initial frame in blue color and (b) representative snapshot after 1 μs simulations in red color. The K-loop, Helix like region (HLR), and helix supporting loop region (HSLR) are specified, with the K-loop shown in licorice representation. SH1 in grey licorice representation. Here, initially residue no 395 to 409 is in proper helix (blue colour helix); over the simulation its in coil (red colour coiled loop; however still closer to helix) form.

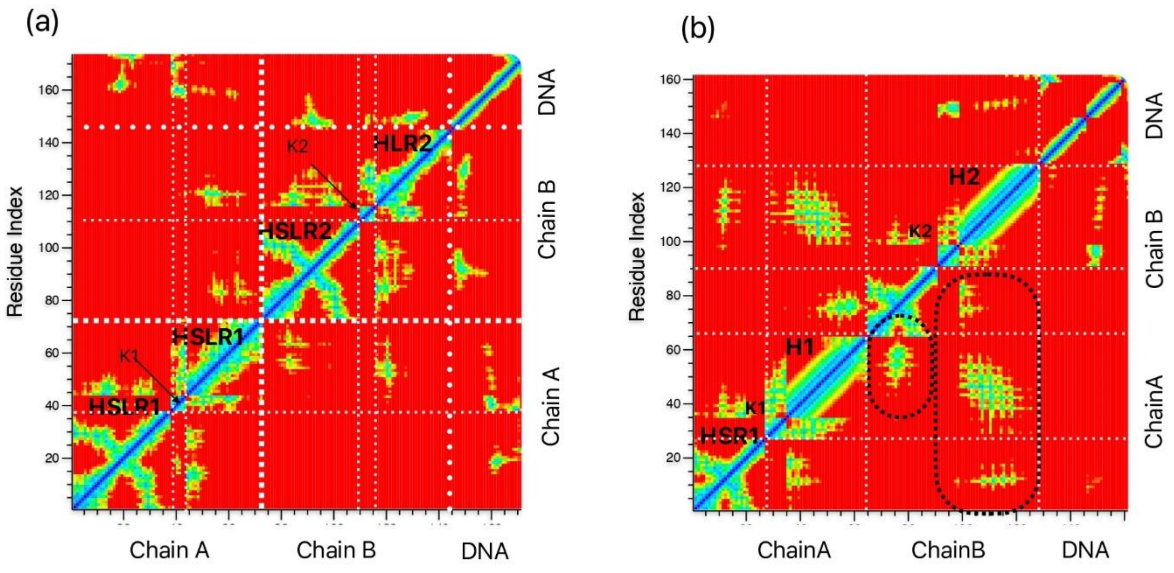

Figure S6: Comparison between a) ScTopoII and b) hTopoII and its inter chain interactions along with DNA. Encircle regions showcasing the major difference between ScTopoII and hTopoII. Here, K2 (chain B) interacts with DNA and K1 (Chain A) loop is not interacting with DNA.

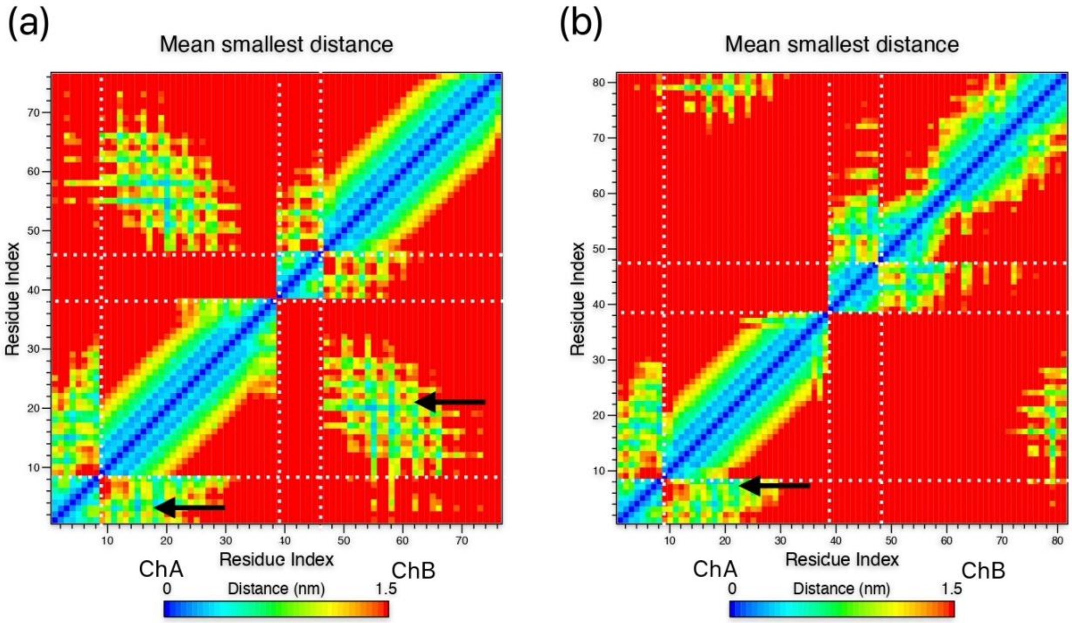

Figure S7: Interaction of Helix vs Helix and Helix vs K-loop region of a) tilted hTopoII (PDB: 6ZY7) and b) symmetric hTopoII (PDB: 6ZY8).

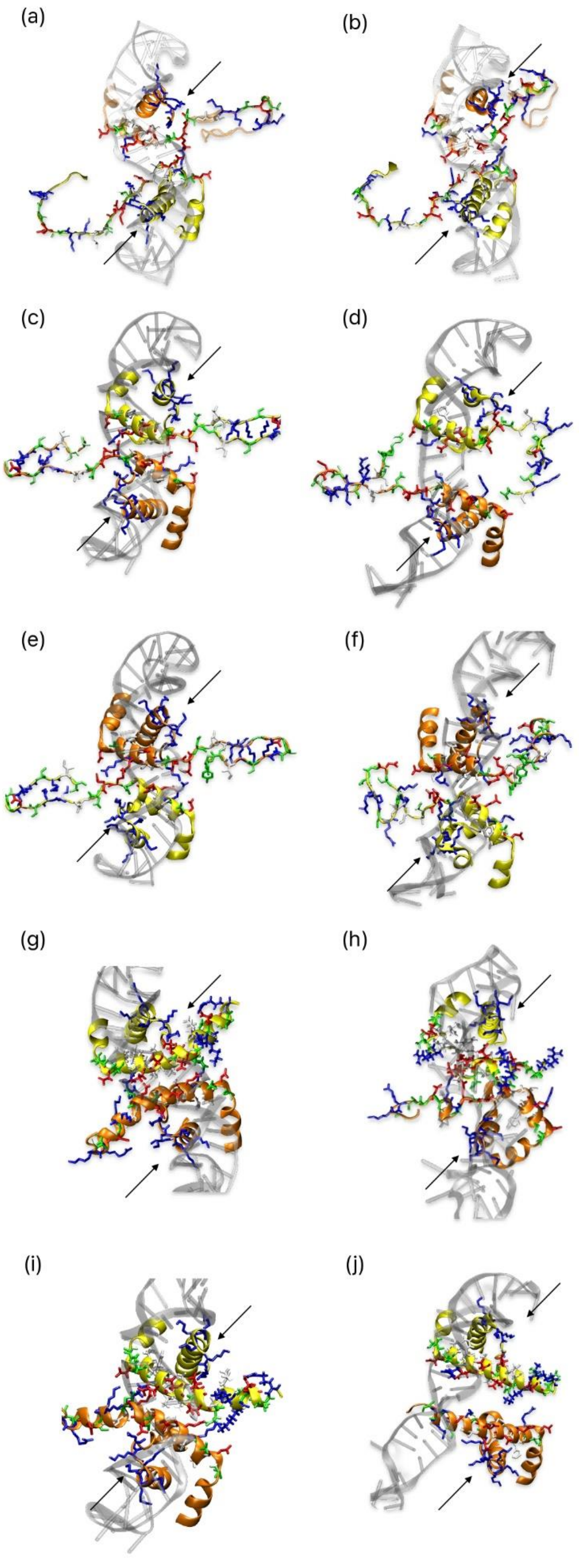

Figure S8: Left side is a representative snapshot of initial structure of ScTopoII and right after 1 μs. a) and b) are the same structures as in Fig 2(e). Subsequently, the HSLR (^{394}T-K^{420}) region was redesigned

using MOE loop modeller software, transitioning from a loop model to a perfect helix, displayed from top to bottom. Additionally, the figure highlights the K-loop region and its interaction with DNA.

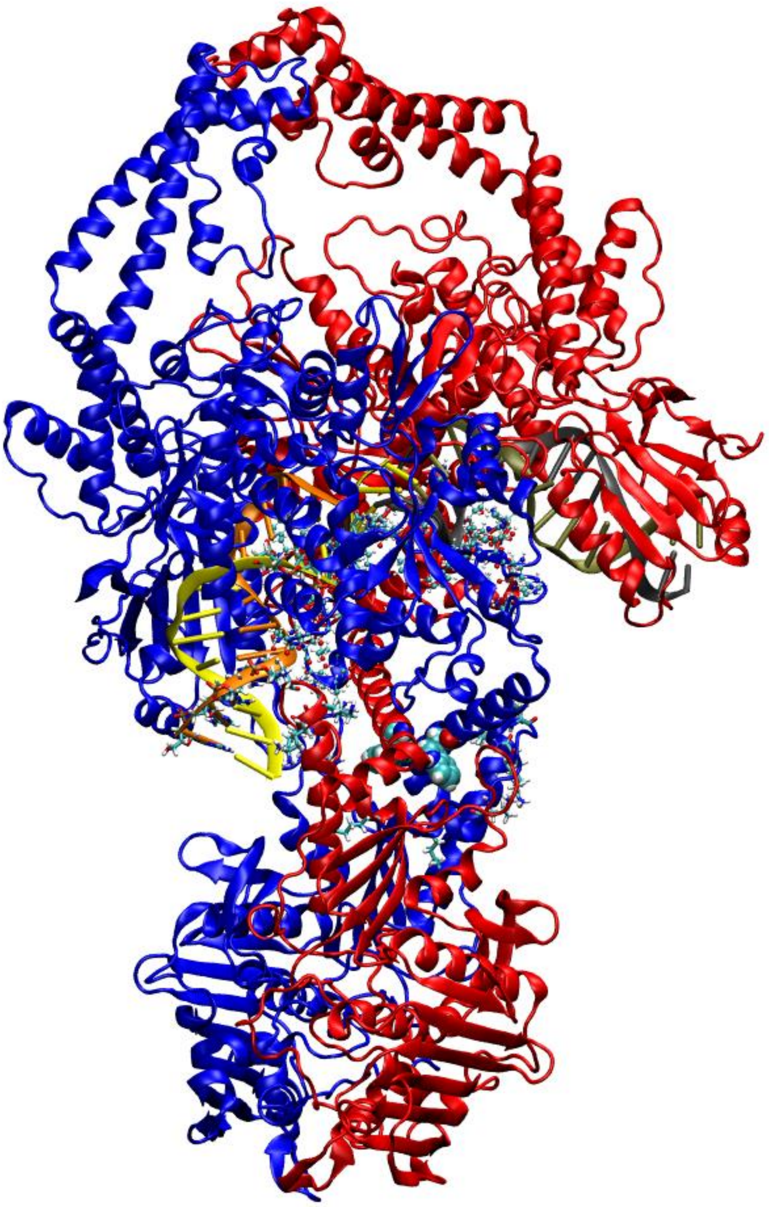

Figure S9: A representative snapshot of entire protein of hTopoII after 1 μs simulations.

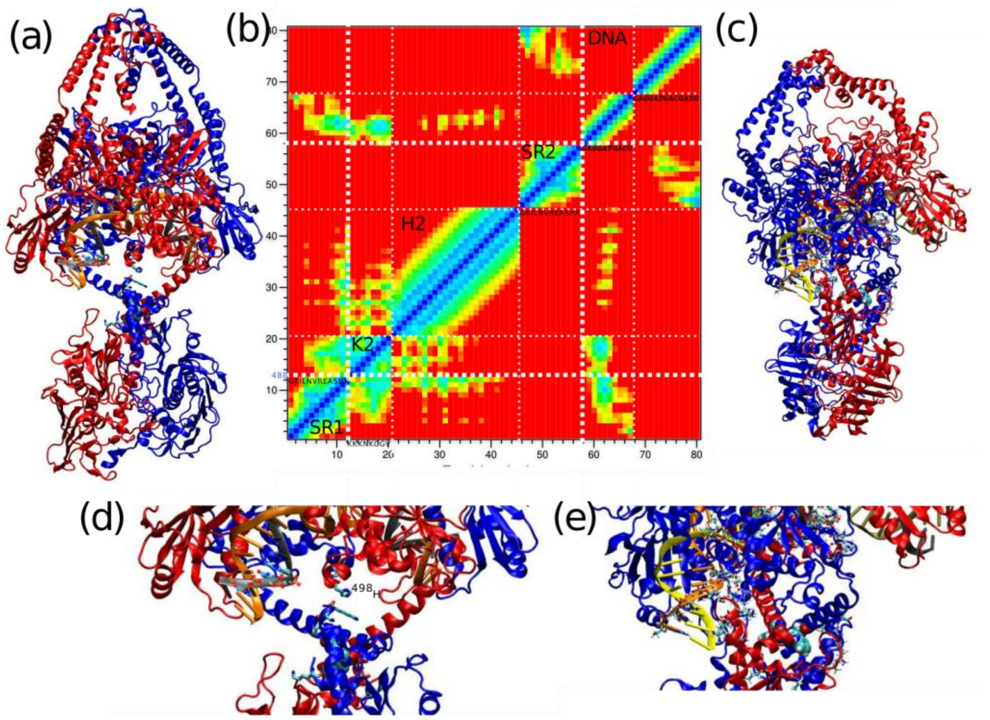

Figure S10: Interaction of Supporting region (SR1; ^{488}GKILNVREASH^{499}K) with K-loop (K2), Helix region (H2), Supporting region (SR2) and DNA (ChainA: GAGGATGACG and ChainB: GAGGATGACGATG); The supporting region is hindering close contacts between K-loop and DNA. a, d) Initial frame from 6ZY7 PDB and c and e) are simulated protein; show casing SR region (^{498}K and K-loop) in liquorice form; Further closer contacts of DNA. b) DCM plot of SR1 and SR2 interactions with other important domains.

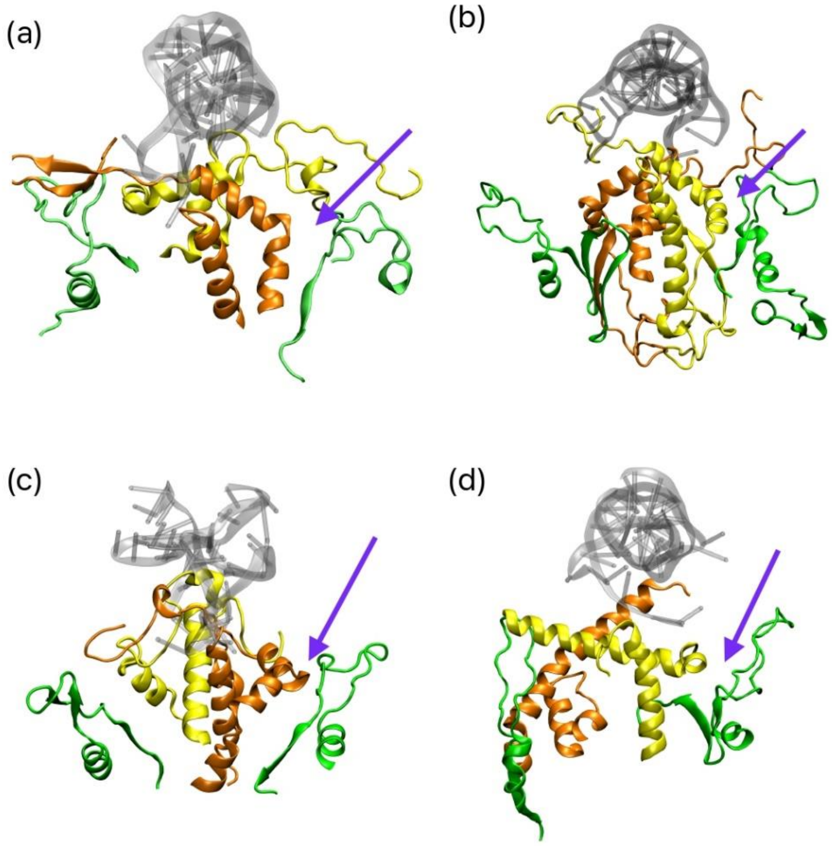

Figure S11: Showcasing probable hotspot in between HSR and HLR of ScTopoII and possible conformations of HSLR in 4 different simulations.

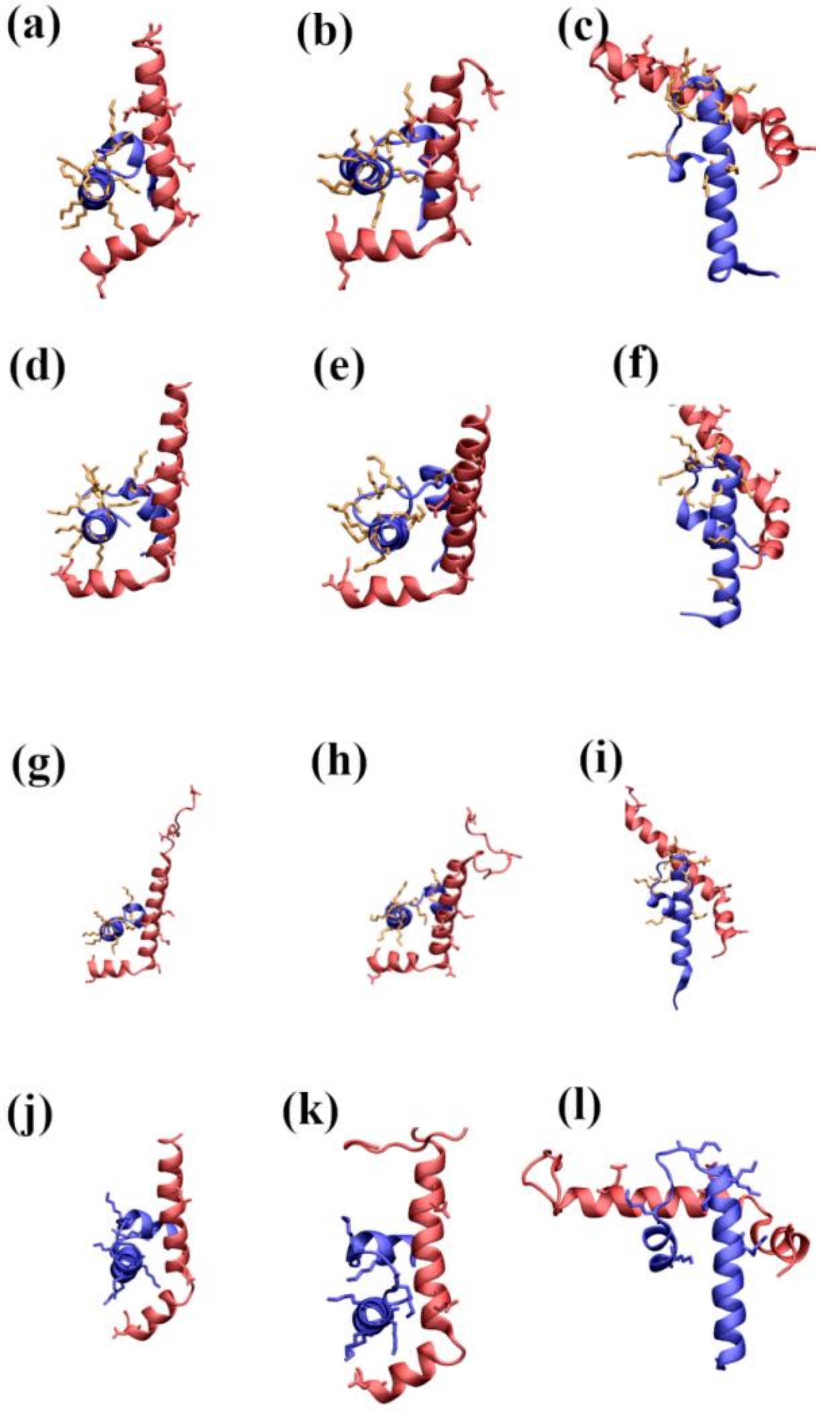

Figure S12: AlphaFold predicted structures for transducer domain of (a-c) ScTopoII (residues 250-450 PDB ID: 4GFH) and corresponding topoisomerases sequences from (d-f) *Candida albicans* (Uniprot ID P87078), (g-i) *Candida glabrata* (Uniprot ID O93794); which are fungal strains compared with (j-l) *Balamuthia mandrillaris* (PDB ID: 7L6S). In each case first column is initial AlphaFold predicted structure and followed by two views of 1µs simulated spectra. In each case the end point of helix is unwinded and interacts with K-loop domain. Intriguing to observe the interaction of location kink in helix domain with respect to K-loop helix. It's interacted in different site in *Balamuthia mandrillaris*. Since, presence of set of R's (we called it as a R-loop) is present in lower region. Refer Figure S1 for the multisequence alignment for these organisms.

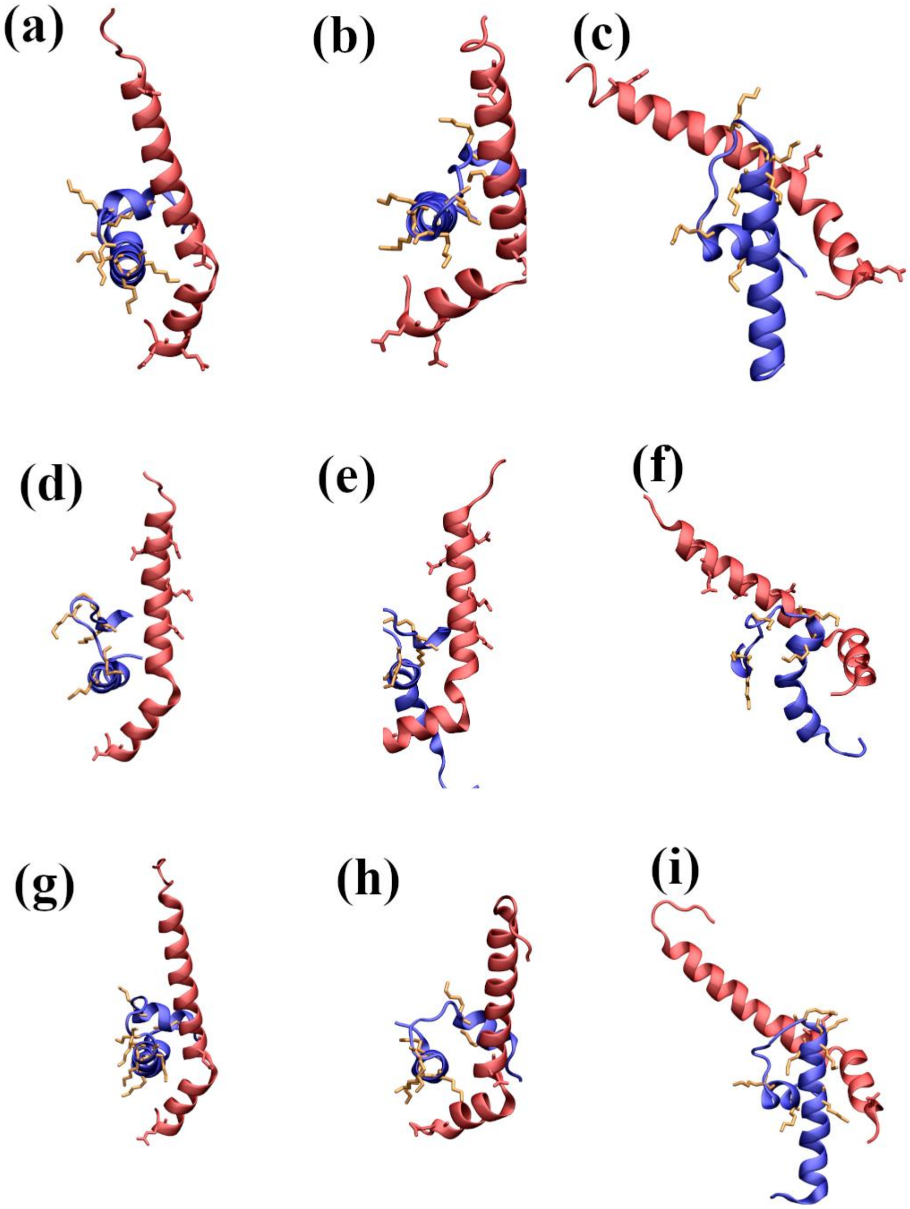

Figure S13: (a-c) *Rozella allomycis*, (d-f) *Capsospora owczarzaki*, (g-i) human Topo II. In each case first column is initial AlphaFold predicted structure and followed by two views of 1µs simulated spectra. In each case the end point of helix is unwinded and interacts with K-loop domain. In case of *Capsospora owczarzaki*, larger deformation in K-loop domain. Refer Figure S1 for the multisequence alignment for these organisms.

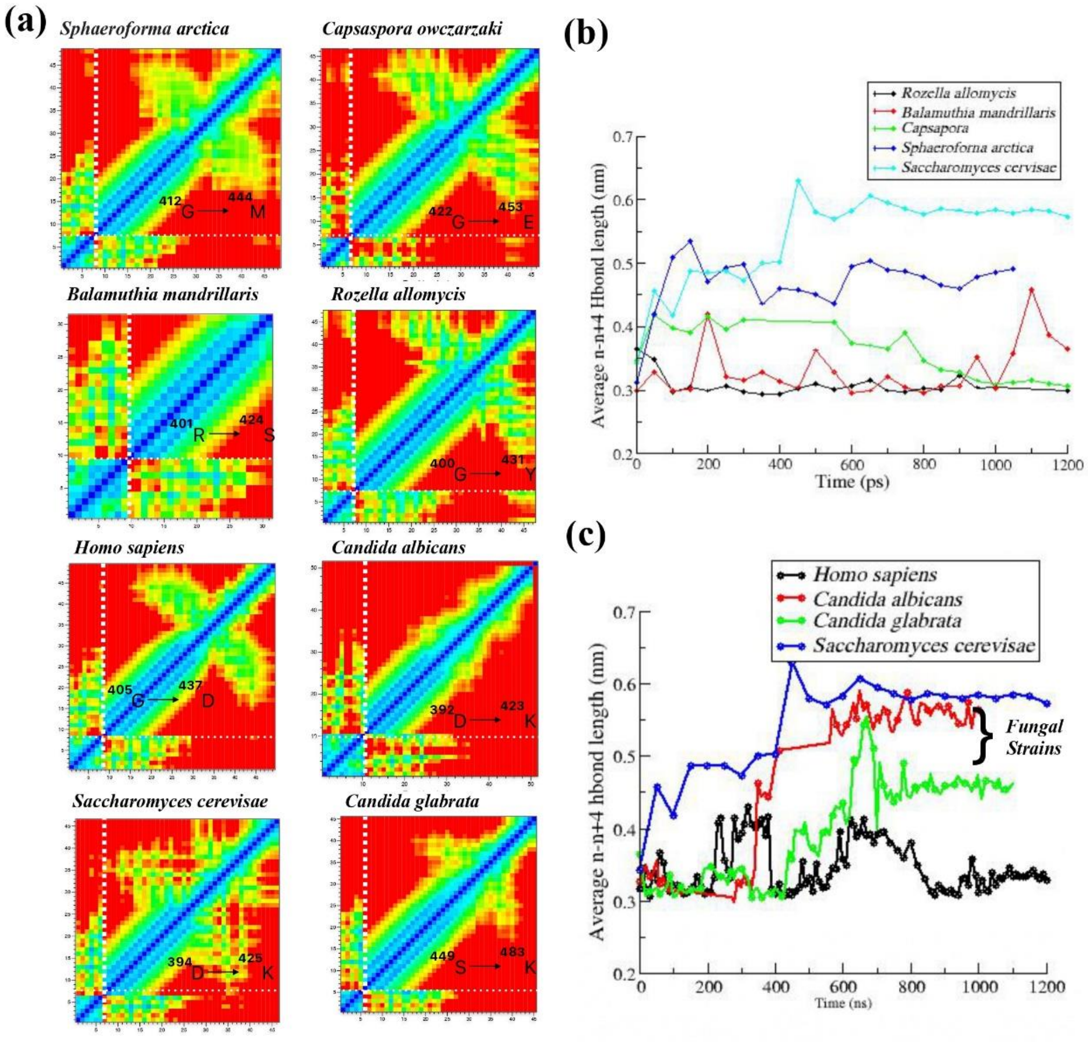

Figure S14: Distance maps comparing AlphaFold-generated and followed by classical MD-simulated structures for 8 organisms, highlighting interactions of the K-loop (R-loop for Balamuthia mandrillaris) and HLR. On the left side, the analysis focuses on average n to n+4 residues H-bond lengths within the alpha-helix region, comparing hTopoII and ScTopoII across the organisms listed in Table S2. On the right side, the length of the helix over the simulations is depicted, indicating convergence to approximately 1.3 nm for ScTopoII, whether generated by Modeller or AlphaFold, whereas hTopoII converges to 3 nm. (Fig. S12 and Fig. S13 for a representative snapshots).

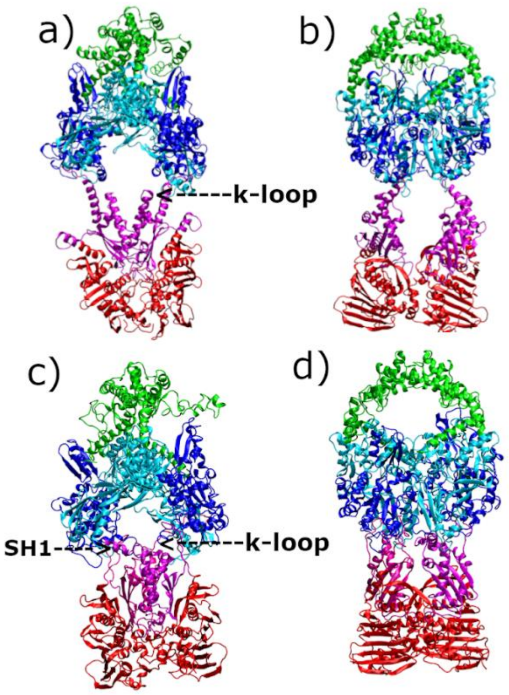

Figure S15: A schematic representation of ScTopoII a) AlphaFold3 generated model[1] with perfect helix, b) rotated view of a). c) Short helix model and d) rotated model of c). Here, we are showcasing location of SH1 and k-loop.

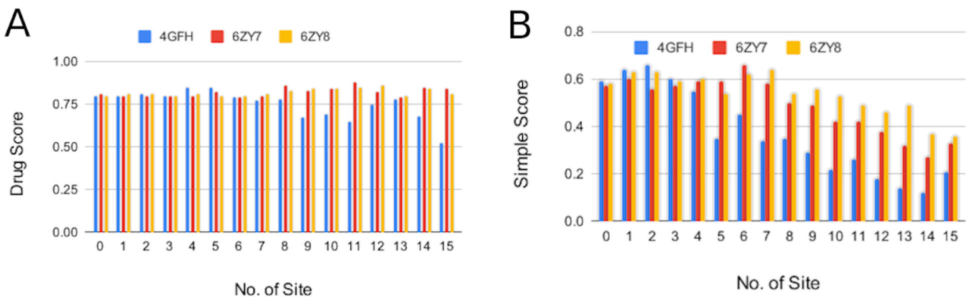

Figure S16: A) Drug score and B) Simple score of the site based on DoGSiteScorer server.

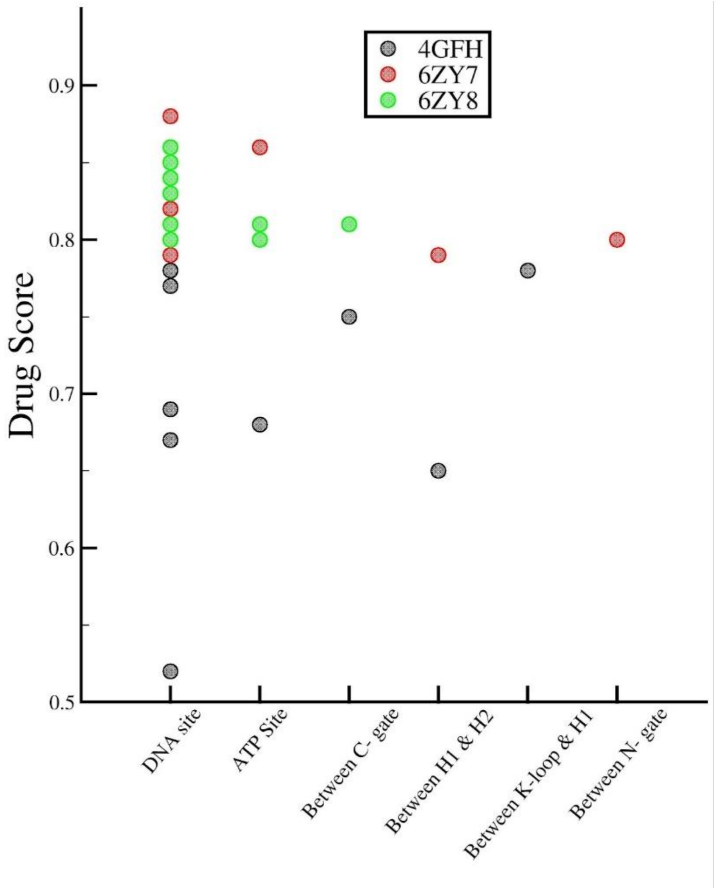

Figure S17: It is a comparison plot between possible sites vs simple site score.

Table S3: Top 30 probable sites generated from MOE for ScTopII with and without DNA.

S. No.	The no. of alpha spheres comprising the site	Rescoring by the Propensity for Ligand Binding index based on amino acid composition of pockets	The no. of Hydrophobic contacts in the receptor	The no. of side chain contact atoms in the receptor	
Site	Size	PLB	Hyd	Side	Residues

1	799	9.98	170	320	1:(ASP322 VAL325 LYS326 SER329 GLU330 LYS333 LYS336 LYS337 SER339 VAL340 LYS341 SER342 ASN410 ALA411 LEU412 LYS413 LYS414 SER415 THR418 SER421 ARG422 ILE423 THR424 TYR426 PRO427 LYS428 LEU429 GLU430 ASP431 GLU449 GLY450 ASP451 SER452 ALA453 LEU454 SER455 LEU456 VAL458 ALA459 LEU461 ALA462 VAL463 GLY465 ARG466 ASP467 ARG475 GLY476 LYS477 ASP526 ASP530 ... LU738 GLN739 SER740 LEU741 ALA742 ALA780)2:(LYS1923 VAL1924 ALA1925 GLN1926 ALA1928 PRO1929 GLY1940 GLU1941 GLN1942 SER1943 LEU1944 ALA1945 ALA1952 GLN1953 ASN1954 PHE1955 VAL1956 TYR1962 LEU1965 PRO1966 ASN1967 GLY1968 ALA1969 PHE1970 GLY1971 THR1972 ARG1973 ALA1974 THR1975 GLY1976 GLY1977 LYS1978 ASP1979 ALA1980 ALA1981 ALA1982 ALA1983 ARG1984 TYR1985 ILE1986 TYR1987 ARG2073 GLY2074 THR211 ... ARG2375 ASP2376 ALA2377 GLU2378 ALA2379)
2	417	4.85	86	183	1:(TYR12 LEU22 LYS23 ARG24 PRO25 ASP26 THR27 GLY30 SER31 VAL32 GLU33 GLN35 PHE152 ARG205)2:(TYR1231 GLU1269 VAL1272 ASN1273 ALA1274 ALA1275 ASP1276 ASN1277 VAL1279 ARG1280 ASP1281 ASN1302 LYS1305 GLY1306 ILE1307 PRO1308 MET1322 ILE1323 PHE1324 THR1329 SER1330 SER1331 ASN1332 TYR1333 ASP1334 ASP1335 ASP1336 GLU1337 LYS1338 LYS1339 VAL1340 THR1341 GLY1342 GLY1343 ARG1344 ASN1345 GLY1346 TYR1347 GLY1348 ALA1349 LYS1350 LEU1351 THR1398 VAL143 ... LYS1570 THR1574 THR1575 ARG1576 ASP1579)
3	334	3.38	78	176	1:(LYS713 ASN714 LEU715 SER717 GLU718 LEU719 LYS720 GLN723 ARG781 TYR782 TYR784)2:(GLU1652 LYS1680 ASP1729 GLN1730 ASP1731 HIS1732 ASP1733 GLY1734 HIS1736 ILE1766 ILE1767 TYR1782 ASN1783 MET1784 PRO1785 TYR1804 TYR1805 LYS1806 LEU1808 GLY1809 THR1810 PRO1868 PHE1886 ARG1893 SER1894 LYS1903 PRO1904 GLY1905 GLN1906 LYS1908 SER1932 GLU1933 ALA1936 TYR1937 HIS1938 HIS1939 GLU1941 SER1943 LEU1944 GLN1946 THR1947 ASP2012 GLU2013 LYS2014 GLU2034 ASP2294 GLU2295 GLU2300)
4	432	2.64	76	151	1:(ASP296 LYS338 LYS341 PHE343 GLN344 ASN347 ASN348 ARG399 MET400 ILE403 ASP405 ALA406 ASN407 GLU408 GLU409 LYS413 TYR426 ARG475 LYS477 MET478 LEU479 ASN480 VAL481 ALA484 SER485 ALA486 GLN488 ILE489 LEU490 LYS491 ASN492 ALA493 ILE495 GLN496 ALA497 LYS499 LYS500 GLY503 LEU504 GLN505 HIS506 ARG507 LYS644)2:(GLU1247 GLU1248 THR1249 ASP1250 CYS1251 ILE1253 LYS1459 SER1460 GLU1462 LYS1463 LYS1464 ARG1465 VAL1497 SER1498 ASP1499 PHE1546 GLN1547 ILE1548 ASN1550 ASN1551 ASP1598 THR1601 ARG1602 ILE1606)

5	343	2.43	55	121	1:(PHE752 GLY765 ALA766 PHE767 GLY768 THR769 ARG770 ALA771 THR772 ASP776 ALA777 THR909 SER910 THR911 ILE912 LYS913 GLU914 MET933 GLU934 GLU935 GLN936 HIS937 ASP938 ASP939 ILE941 GLU1175 ALA1176)2:(LYS1616 SER1618 ASP1619 GLY1620 THR1621 ARG1622 PRO1630 LYS1631 LEU1632 GLU1633 ASP1634 ALA1635 ASN1636 ASP1654 LEU1657 SER1658 VAL1661 ALA1662 LEU1664 ALA1665 ARG1669 ASP1670 LEU1812 ALA1813 GLN1814 ARG1817)
6	254	2	51	112	1:(ASP26 THR27 ILE29 GLY30 SER31 THR34 GLN35 GLU36 GLN37 LEU38 GLN39 TRP40 GLU51 ASN53 ILE56 PRO58 GLY59 PHE61 LYS62 ASP65 VAL218 ARG221 ARG222 ASP225 TYR250 TYR254 GLN300 ILE302 PHE304 ALA309 THR310 THR311 MET312 SER364 THR366 LYS367 GLU368)2:(THR1518 ASN1521 TYR1522 ALA1564 PHE1565 THR1566 SER1567 GLN1568 GLN1572 LEU1573 THR1574 THR1575 ARG1576 VAL1577 LYS1578)
7	126	1.45	44	74	2:(PRO1896 ASP1900 GLY1901 PHE1902 LYS1903 GLN1906 GLY1950 LEU1951 ALA1952 GLN1953 ASN1954 PHE1955 GLY1957 SER1958 ASN1959 ILE1961 TYR1962 LEU1963 THR1975 GLY1976 GLY1977 LYS1978 MET2027 ILE2028 VAL2030 ASN2031 GLY2032 ALA2033 GLU2034 TYR2043 ILE2044 PRO2045 PRO2046 PHE2072 MET2089 ALA2108 ARG2109 TRP2111 ILE2173)
8	182	1.04	44	84	1:(ILE523 MET524 THR525 ASP526 GLN527 ASP528 HIS529 GLY531 SER532 LYS535 GLU558 PHE559 ILE560 THR561 ILE563 TYR579 ASN580 MET581 PRO582 TYR584 TYR601 LYS625 THR661 VAL662 LEU663 ASP664 LEU667 ILE670 ASP674 PHE675 LYS678 GLU679 LEU682 HIS735 GLN807 GLU808)
9	52	0.84	26	36	1:(PRO842 PHE844 GLN864 MET865 HIS866 PRO867 GLU878 PRO881 LEU882 ARG883 TYR884 ILE970 SER971 LEU972 VAL990)
10	194	0.83	46	89	1:(PHE299 GLN301 THR311 MET312 GLY313 GLY314 THR315 VAL317 ASN318 TYR319 THR321 ASP322 LYS346 PHE362 THR363 GLN369 LEU370 THR371 ARG373 VAL374 LYS375)2:(ASP1229 ILE1232 GLY1233 SER1234 THR1237 GLN1238 GLU1239 GLN1240 GLN1242 THR1258 ILE1259 PRO1261 PHE1264 LYS1265 VAL1421 THR1514 MET1515 THR1569 GLU1571)
11	114	0.64	35	59	2:(GLN1240 LEU1241 GLN1242 TRP1243 GLU1254 PHE1264 VAL1421 ARG1424 ARG1425 ASP1428 PHE1502 GLN1503 GLN1504 ILE1505 SER1506 PHE1507 ALA1512 THR1513 THR1514 MET1515 PHE1553)
12	66	0.62	26	45	1:(GLN527 ASP528 ILE563 TYR601 TYR602 LYS603 SER729 GLU730 ALA733 TYR734 HIS735 GLU808 LYS811)2:(GLU1921 LEU1922 LYS1923 GLN1926 TYR1987)
13	87	0.49	24	41	1:(PRO693 ASP697 GLY698 PHE699 LYS700 GLN703 GLY747 LEU748 ALA749 GLN750 ASN751 PHE752 SER755 ASN756 ILE758 TYR759 LEU760 MET824 ILE825 ASN828 GLY829 ALA830 GLU831 GLY832)

14	73	0.43	28	42	1:(LEU654 ARG655 TYR657 GLU658 PRO659 LEU685 ILE689 ILE692 PRO693 ASN694 PHE699 LYS700 PRO701 ARG704 GLU816 TRP817 PHE978 ASP979 PRO980 HIS981 GLY982 LYS983 ILE984)
15	49	0.4	13	34	1:(GLU66 VAL69 ASN70 ASP73 ARG77 ILE104 ILE120 SER127 SER128 THR138 GLY139 GLY140 ASN142 GLY143 TYR144 GLY145 ALA146 LYS147 SER307 ILE308)2:(TYR1215)
16	96	0.4	25	54	2:(ARG1791 GLU1792 SER1795 HIS1796 LYS1797 PHE1798 THR1799 TRP1800 GLN1802 ILE2009 GLN2010 GLU2011 LYS2014 THR2289 SER2290 ASP2294 SER2297 SER2298 HIS2299 GLU2300 ASP2301 GLU2303 ILE2306 ASN2307)
17	53	0.37	24	51	1:(ARG1045 ASN1046 ILE1049 GLN1050 GLU1053 ARG1059 ASP1073 GLU1074 ILE1075 GLN1078 GLU1108 THR1112 TYR1113 GLU1114)2:(TRP2325)
18	76	0.33	20	42	1:(ASN1072 ASP1073 GLU1074 ILE1075 ALA1076 ILE1079 ASP1081 VAL1082 ALA1085 GLU1089 GLU1090 ASP1091 SER1094 SER1095 HIS1096 GLU1100 ILE1103 ASN1104 GLY1105 PRO1106 GLU1107 GLU1108)
19	53	0.33	20	35	1:(PHE559 ILE560 THR561 PRO562 LYS565 THR575 ILE576 ALA577 TYR579 GLU615 TYR616 PHE617 SER618 ASN619 LEU620 HIS623)
20	65	0.33	25	47	1:(PRO562 ILE563 ILE564 LYS565 LYS600 TYR601 TYR602 LYS603 GLY604 THR607 SER608 LEU609 GLU612 GLU615 TYR616)2:(GLU1921 ASN1967)
21	87	0.32	13	37	2:(ILE1767 MET1784 PRO1785 TYR1787 GLU1788 ARG1791 GLN1802 TYR1804 ASP2294 GLU2295 GLU2296 SER2297 SER2298 HIS2299 GLU2300)
22	16	0.29	14	30	1:(THR27 TYR28 ASN142 TYR144 GLN365)2:(HIS1223 ARG1227 THR1230 TYR1231)
23	80	0.29	33	54	1:(TYR782)2:(THR1651 GLU1652 GLY1653 ASP1654 SER1655 ALA1656 LEU1659 MET1727 THR1728 ASP1729 GLN1730 PHE1762 ILE1763 THR1764 ILE1766 TYR1805 LYS1806 GLY1807 LEU1808 VAL1816)
24	66	0.27	15	39	1:(LYS82 ASN227 VAL230 ARG231 ASP232 ILE233 ASN234 VAL235 LEU242 ILE244 ARG245 ASN246 PHE247 ARG283 GLU289 ASN354)
25	17	0.22	11	24	1:(ILE564 TYR584 ARG588 TRP597 GLN599 TYR601 GLN807 GLU808 ASP809 GLU810)
26	74	0.19	21	42	2:(ARG2248 ASN2249 ALA2250 ILE2251 ILE2252 GLN2253 GLU2256 ARG2262 SER2273 PRO2274 ASP2276 ALA2279 GLU2280 ILE2282 ASN2283 GLY2314 THR2315)
27	10	0.17	5	18	1:(HIS109 LYS111 GLU112)2:(ASP1213 LYS1214 TYR1215 GLN1216 LYS1217)
28	61	0.16	14	34	1:(ASP528 HIS529 ALA686 ARG690 PRO701 LYS705 THR732 ALA733 TYR734 HIS735 HIS736 GLU808 VAL813 GLU814)

29	45	0.15	21	41	2:(GLN1730 ASP1731 GLY1734 SER1735 LYS1738 GLU1761 ILE1763 ASN1783 PRO1785 LYS1828 TYR1860 VAL1865 LEU1866 ASP1867 PRO1868 PHE1878 LYS1881 GLU1882 LEU1885)
30	71	0.12	34	53	1:(GLU268 ASP269)2:(LEU1648 LEU1650 TYR1675 PRO1676 LEU1677 ARG1678 GLY1679 LYS1680 MET1681 LEU1682 GLU1697 ILE1698 ILE1701 MET1705 LEU1724 ILE1741 PHE1744)
With DNA					
Site	Size	PLB	Hyd	Side	Residues
1	3198	11.21	553	1206	1:(ARG262 LEU264 ASP265 ASN266 GLY267 GLU268 ASP269 GLY270 ALA272 SER274 ASP275 ILE276 LYS327 SER329 GLU330 ILE331 LEU332 LYS333 LYS334 LYS335 LYS336 LYS337 LYS338 SER339 VAL340 LYS341 GLN344 LEU385 ASN389 MET392 LYS393 THR394 ASP395 LEU396 ALA397 THR398 MET400 PHE401 GLU402 ILE403 ALA404 ASP405 ALA406 ASN407 GLU408 GLU409 ASN410 ALA411 LEU412 LYS413 ... LY765 ALA766 ARG781 TYR782 ILE783 TYR784)2:(LYS1438 ARG1439 GLN1440 ASP1442 ASP1473 ILE1474 GLN1521 ILE1522 ASN1524 ASN1525 ASP1572 THR1575 ARG1576 MET1577 GLU1579 ILE1580 LYS1605 GLU1626 SER1629 ALA1630 LEU1633 PRO1650 LEU1651 ARG1652 GLY1653 LYS1654 MET1655 LEU1656 ASN1657 VAL1658 ARG1659 GLU1660 ALA1663 ASP1664 GLN1665 ILE1666 LEU1667 LYS1668 ASN1669 ILE1672 GLN1673 ILE1675 LYS1676 MET170 ... ASP2350 ALA2351 GLU2352 ALA2353 ARG2354)3:(DG11 DC11 DG11 DG11 DT11 DA11 DG11(DC11) DA11 DG11 DT12 DA13 DG14 DG15)4:(DC2 DT3 DA4 DC5 DT6 DG7 DC8 DT9 DA10 DC11 DC11! DG11 DC11 DG11 DG11)
2	1088	3.12	176	483	1:(ASN480 VAL481 GLU483 ALA484 SER485 ALA486 ASP487 GLN488 ILE489 LEU490 LYS491 ASN492 LYS644 LYS651 PRO693 GLY698 PHE699 LYS700 GLN703 GLN743 THR744 ILE746 GLY747 LEU748 ALA749 GLN750 ASN751 PHE752 GLY754 SER755 ASN756 ILE758 TYR759 LEU760 LEU761 PRO763 PHE767 THR772 GLY773 GLY774 LYS775 ALA777 ALA778 ILE783 MET824 ILE825 ASN828 GLY829 ALA830 GLU831 ... E970 SER971 ASN974 VAL976 LYS986 ARG1177)2:(ASN1443 GLU1445 ASP1446 GLY1447 SER1451 LYS1510 LYS1511 LYS1512 LYS1513 LYS1514 ASN1566 MET1569 LYS1570 PHE1578 GLU1579 ILE1580 ALA1581 ASP1582 ALA1583 ASN1584 GLU1585 GLU1586 ASN1587)3:(DC1 DC2 DT3 DA4 DC5 DT6 DG7 DC8 DT9 DA10 DC11)4:(DG11 DG11 DT11 DA11 DG11(DC11) DA11 DG11 DT12 DA13 DG14 DG15)

3	530	1.7	127	262	1:(TYR28 LYS62 ASP65 GLU66 VAL69 ASN70 ASP73 ASN74 ARG77 ILE104 ILE120 PHE121 THR126 SER127 SER128 ASN129 ASP132 THR138 GLY139 GLY140 ARG141 ASN142 GLY143 TYR144 GLY145 ALA146 LYS147 ARG222 PHE299 GLN301 VAL305 ASN306 ILE308 ALA309 MET312 GLY313 GLY314 THR315 VAL317 ASN318 THR321 LYS346 ASN359 PRO360 ALA361 PHE362 THR363 SER364 GLN365 THR366 LYS367 GL ... HR371 THR372 ARG373 VAL374 LYS375 ASP376)2:(TYR1189 ARG1201 ASP1203 THR1204 TYR1205 ILE1206 GLY1207 SER1208 VAL1209 GLU1210 GLN1212 GLN1216 ILE1233 PRO1235 PHE1238 LYS1239 PHE1241 ASP1242 GLU1243 VAL1246 VAL1395 MET1396 ARG1399 ILE1485 ALA1486 THR1487 THR1488 MET1489 SER1541 GLN1542 THR1543 LYS1544 GLU1545)
4	326	1.18	79	146	2:(LYS1654 THR1702 ASP1703 GLN1704 ASP1705 HIS1706 ASP1707 GLY1708 SER1709 HIS1710 ILE1740 TYR1756 ASN1757 MET1758 TYR1778 TYR1779 LYS1780 PHE1860 ALA1863 ASP1864 ILE1866 ARG1867 ILE1869 PRO1870 ASN1871 PHE1876 LYS1877 PRO1878 GLY1879 ARG1881 LYS1882 THR1909 ALA1910 TYR1911 HIS1912 HIS1913 GLY1914 GLU1915 SER1917 LEU1918 THR1921 LYS1981 ILE1983 GLN198 ... PRO1992 GLU1993 TRP1994 PHE2155 PRO2157)4:(DA10 DC11)
5	365	0.87	96	173	1:(ILE29 GLY30 GLN35 GLU36 GLN37 LEU38 GLN39 TRP40 ILE56 PRO58 PHE61 LYS62 ASP65 VAL218 ARG221 ARG222 ASP225 TYR250 SER298 PHE299 GLN300 GLN301 ILE302 SER303 PHE304 ALA309 THR310 THR311 MET312 GLY313 PHE343 LYS346 ASN347 PHE350 THR363 THR366 LYS367 GLU368 GLN369)2:(PHE1476 GLN1478 MET1489 GLY1490 GLY1491 THR1492 VAL1494 ASN1495 THR1498 ASP1499 VAL1502 LYS1503 SER1519 PHE1520 LYS1523 PHE1539 THR1540 GLN1546 LEU1547 THR1548 ARG1550 VAL1551 LYS1552)
6	172	0.55	40	89	1:(THR448 GLU449 GLY450 ASP451 SER452 ALA453 MET524 THR525 ASP526 GLN527 ASP528 ASP530 THR561 PRO562 ILE563 TYR602 LYS603 GLY604 LEU605 GLY606 TYR616 PHE617 HIS735 HIS736 GLY737 GLU738 GLN739 SER740)2:(LYS1897 ALA1956 ARG1958 TYR1959)3:(DC11 DC11 DG11)
7	267	0.44	55	115	1:(LYS712 PRO797 ALA798 ASP800 PRO801 LEU802 TYR803 LYS804 TYR805 ILE806 GLN807 GLU808 LYS811 VAL813 GLU814 PRO815 GLU816 LYS1007 ARG1008 ASP1010 HIS1011 MET1012 ARG1015 LEU1016 TRP1018 GLU1019 ASN1061 LYS1062 GLU1063 GLY1064 LYS1065 ASN1072 ASP1073 GLU1074 ILE1075 ALA1076 GLU1077 ILE1079 ASN1080 ASP1081 VAL1082 LYS1083 GLY1084 THR1086 GLU1093 SER1094 SER1095)

8	201	0.4	47	104	1:(MET1 GLU4 LYS11 TYR12)2:(GLU1243 VAL1246 ASN1247 ALA1248 ALA1249 ASP1250 ASN1251 ARG1254 MET1258 ASN1276 ASP1277 GLY1278 LYS1279 GLY1280 ILE1281 PRO1282 ILE1297 PHE1298 SER1304 SER1305 ASN1306 ASP1308 LYS1313 THR1315 GLY1317 ARG1318 ASN1319 GLY1320 TYR1321 GLY1322 ALA1323 ASP1339 ASN1341 THR1372 VAL1374 SER1484 LYS1544)
9	149	0.28	50	90	1:(ILE15 GLN17 HIS20 ILE21 ARG24 THR27 TYR28 LEU125 TYR144)2:(GLN1194 LEU1195 HIS1197 ILE1198 TYR1205 ILE1206 LYS1239 GLU1243 HIS1300 LEU1301 LEU1302 THR1303 SER1304 TYR1307 ARG1318 ASN1319 GLY1320 TYR1321 GLY1322 LYS1324 LEU1325 GLN1542 THR1543 LYS1544)
10	178	0.22	40	75	1:(ALA766 PHE767 GLY768 ALA771 THR772 GLY773 GLY774 LYS775 ASP776 ALA777 TRP908 THR909 SER910 THR911 LYS913 GLU914 HIS937 ASP939 ASN940 ILE941)2:(ASN1584 GLU1585 PRO1604 LYS1605 LEU1606 GLU1607 ASP1628 LEU1631 SER1632 VAL1635)4:(DC11 DG11 DG11)
11	133	0.19	40	71	1:(ASN751 PHE752 VAL753 TYR759 LEU762 THR769 ARG770 ALA771 THR772 GLY773 TRP872 GLU902 LEU903 PRO904 THR907 TRP908 SER910 ASP938 ASP939 ASN940 ILE941 LYS942 ARG1172 ASP1173 ALA1174 GLU1175 ALA1176 ARG1177)2:(LYS1590 LYS1591 SER1592 ASP1593)
12	57	0.14	25	36	1:(ASP296 ILE297 SER298 VAL340 GLN344 ILE345 ASN347 ASN348 ARG399 MET400 PHE401 GLU402 ILE403 ALA404)2:(LYS1518 SER1519 PHE1520 GLN1521)
13	97	0.12	28	58	1:(GLU449 ILE523 MET524 THR525 ASP526 GLN527 ASP528 HIS529 GLY531 SER532 ILE534 LYS535 GLU558 PHE559 ILE560 THR561 PRO562 ILE563 TYR579 ASN580 MET581 PRO582 LYS678 GLU679 LEU682)
14	107	0.09	24	48	1:(VAL69 ALA72 ASP73 LYS75 VAL76 PRO79 VAL137 THR138 GLY139 SER229 VAL230 ARG231 ASP232 ILE233 TYR281 ARG283 ASN285 ASN286 ARG287 TRP288 GLU289 VAL305 ASN306 SER307 ASN354 CYS355 LEU356)
15	89	0.09	27	47	1:(LYS499 LEU504 GLN505 HIS506 LYS508 LYS509 TYR510 GLU511 PHE541 SER544 SER545 PHE546 PRO547 GLY548 LEU549)2:(ARG1439 LEU1441 ASP1442 ALA1449 LYS1450 SER1451 ASP1452 ILE1453)
16	118	0.07	41	60	2:(THR1702 GLN1704 SER1709 HIS1710 ILE1711 LYS1712 GLY1713 GLU1735 PHE1736 ILE1737 HIS1800 LEU1801 LYS1802 PHE1804 HIS1805 SER1806 LEU1807 TYR1814 LEU1844 LYS1845 GLU1846 ILE1847 PRO1848 ILE1849 PHE1852 GLU1856 PHE1860)
17	37	0.06	14	33	1:(TRP40 GLU51 SER257 LEU258 GLU259 LYS261 VAL294 SER295 ASP296 ILE297 SER298 GLN300 PHE350 ARG399)
18	52	0.03	16	31	1:(SER485 ALA486 ASP487 GLN488 ILE489)2:(ARG1439 GLN1440 LEU1441 ASP1442 ASN1443 GLY1444 GLY1447 ALA1448 MET1569 LYS1570 THR1571 ASP1572 ALA1574 THR1575)

19	59	0.02	25	52	1:(TRP1122)2:(ARG2222 ASN2223 ILE2226 ARG2236 GLU2251 PRO2283 GLU2284 GLU2285 GLY2288 THR2289 TYR2290 GLU2291)
20	94	0.01	13	36	2:(ARG1765 GLU1766 GLU1767 SER1769 HIS1770 PHE1772 THR1773 TRP1774 ASN2249 ASP2250 ALA2253 GLU2254 GLN2255 THR2276 ASN2278 VAL2279 ILE2280 ASN2281 GLY2282 PRO2283 TYR2287)
21	161	0.01	36	96	1:(ALA722 GLN723 PRO726 GLU738 GLN739 ALA742 GLN743 ILE746 ALA778 ALA779 ALA780 ARG781 TYR782)2:(ALA1899 ALA1902 PRO1903 SER1906 GLY1914 GLU1915 GLN1916 LEU1918 ALA1919 ALA1957 ARG1958)3:(DC11 DC11 DG11 DC11)4:(DC11 DC11 DG11 DC11 DG11 DG11)
22	71	0.01	18	37	2:(LYS1252 VAL1253 PRO1256 ASN1404 GLY1405 SER1406 VAL1407 ARG1408 PHE1424 ARG1460 ASN1462 ASN1463 ARG1464 TRP1465 GLU1466 PHE1481 VAL1482 ASN1483 SER1484 ILE1485 ASN1531 CYS1532 LEU1533)
23	46	-0.01	20	36	1:(ILE689 ILE692 PRO693 ASN694 ASP697 PHE699 LYS700 PRO701 ARG704 LYS804 GLU816 TRP817 PHE978 PRO980)
24	29	-0.01	20	31	1:(TYR130 ASP131 ARG141 ASN142)2:(ILE1192 SER1193 HIS1197 ILE1198 LEU1199 LYS1200 ARG1201 THR1204 TYR1205)
25	74	-0.03	22	36	1:(TYR472 PRO473 LEU474 ARG475 LYS477 MET478 LEU479 ASN492 ALA493 GLU494 ILE495 ILE498)2:(ASP1442 ASN1443 GLY1444)4:(DT11&)
26	46	-0.03	19	33	2:(TYR1219 ASP1220 GLU1221 ASP1224 ARG1397 TYR1401 LEU1414 LYS1417 SER1418 LEU1419 LYS1420)
27	61	-0.04	27	48	2:(LEU1215 GLN1216 TRP1217 GLU1228 VAL1395 ARG1398 ARG1399 ASP1402 GLN1477 GLN1478 ILE1479 SER1480 PHE1481 ALA1486 THR1487 THR1488)
28	81	-0.04	18	45	1:(LYS23 ARG24 PRO25 ASP26 GLY30 SER31 VAL32 GLU33)2:(TYR1307 ASP1309 ASP1310 GLU1311 LYS1312 LYS1313 VAL1314 THR1315 GLY1317 ARG1318 ASN1483 GLU1535 ASN1536 PRO1537)
29	22	-0.07	21	26	2:(ILE1716 GLU1720 LEU1727 LEU1807 GLN1808 ASP1811 LYS1812 ASP1813 TYR1814 ILE1815 ASP1816 PHE1819)
30	44	-0.07	19	28	1:(ILE1033 LYS1036 LEU1038 THR1039 VAL1040 THR1041 LEU1124)2:(ILE2210 LYS2213 LEU2215 THR2216 VAL2217 THR2218 LEU2301)

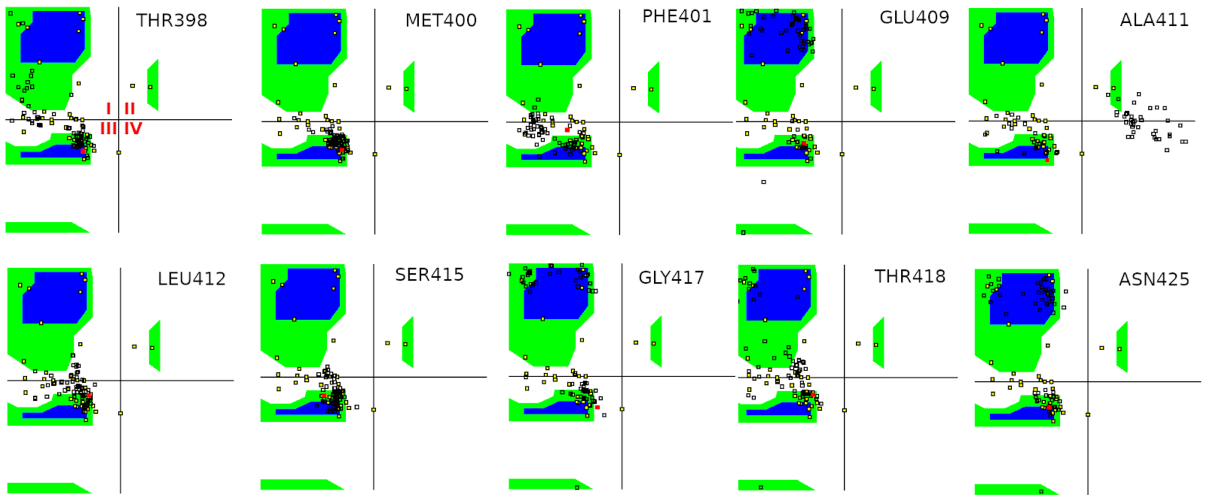

Figure S18: **Ramachandran plot of selected residues in the HLR region of ScTopoII.** The plot illustrates the φ (phi) and ψ (psi) torsion angles of the residue i+1 and the corresponding amino acid at i+2 sampled over 60 frames from the MD simulation, with a step size of 200 ns. Red dots indicate the ideal φ and ψ values for each residue. This analysis highlights the conformational stability and structural fidelity of the HLR region during the simulation.

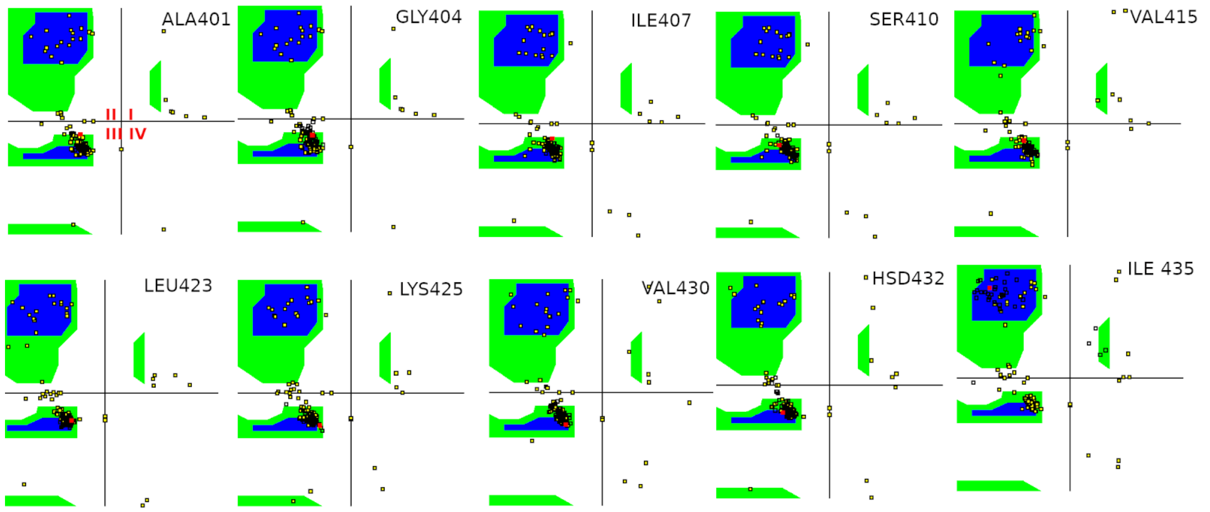

Figure S19: **Ramachandran plot of selected residues in the helix (H1 and H2) region of hTopoII.** The plot illustrates the φ (phi) and ψ (psi) torsion angles of the residue i+1 and the corresponding amino acid at i+2 sampled over 60 frames from the MD simulation, with a step size of 200 ns. Red dots indicate the ideal φ and ψ values for each residue. This analysis highlights the conformational stability and structural fidelity of the HLR region during the simulation.

Analysing of ERRAT Plots: we computed overall quality factors of our models, compared with AlphaFold3 predicted (model 03) and hTopoII. Further, we compared time evolution of overall quality factor of our models.

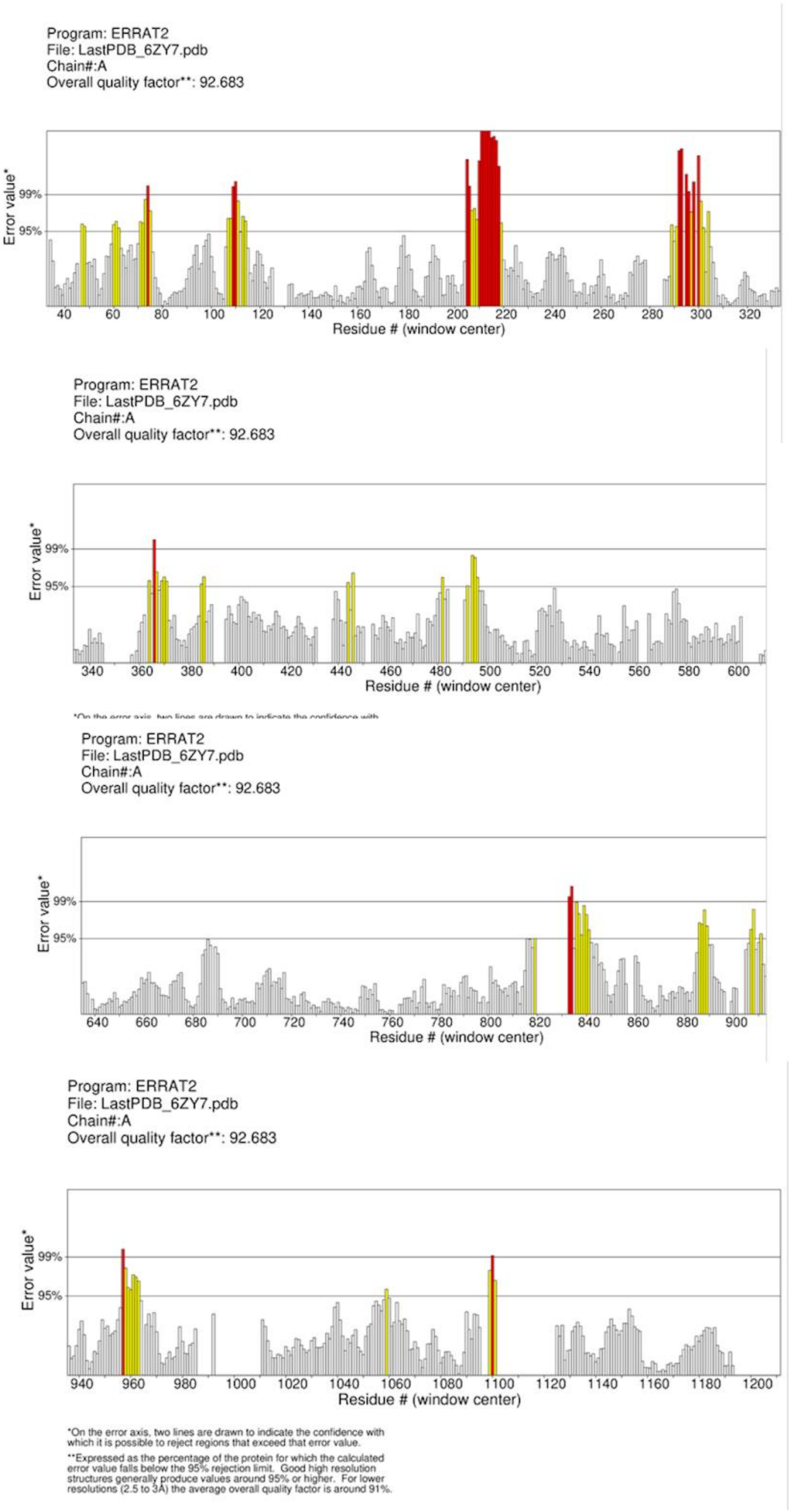

Figure S20: Result of ERRAT plot of hTopoII.

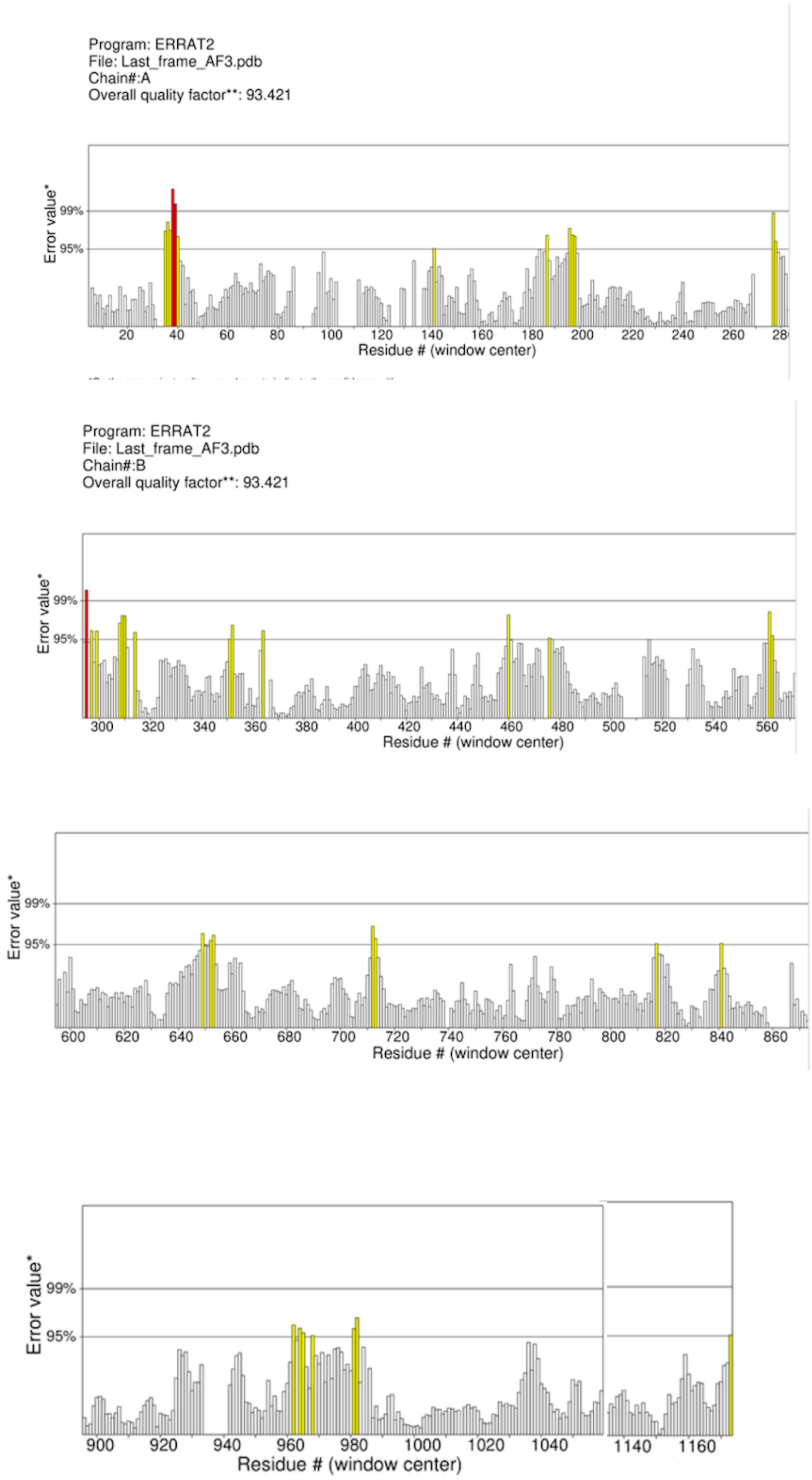

Figure S21: Result of ERRAT plot of ScTopoII. The right part of the last panel was taken from next page and just appended for saving length of the figure.

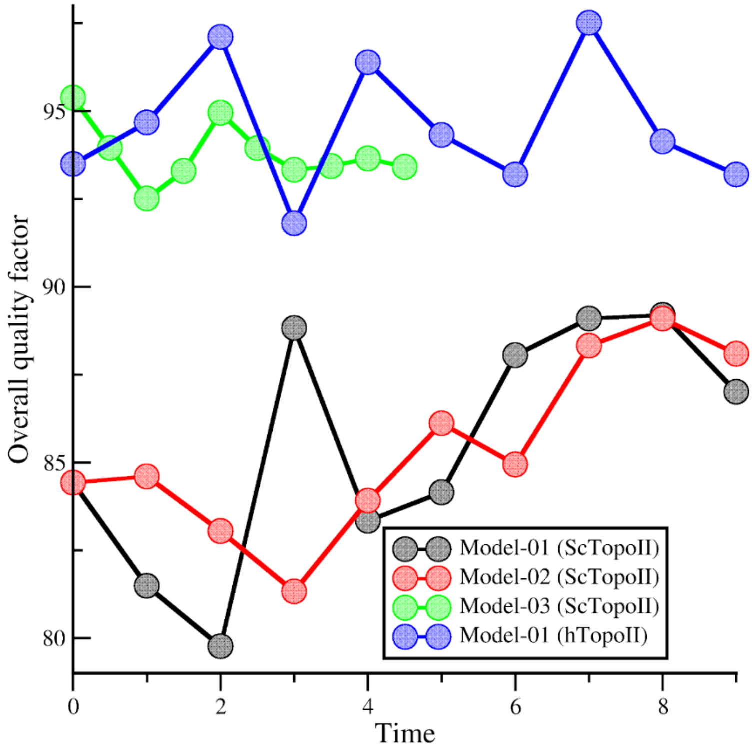

Figure S22: Analysis of overall quality factor with respect to simulation time. Over the time, we observed our models (01 and 02) of ScTopoII saturates at 90% overall quality factor. However, AlphaFold3[1] generated over the time its decreasing its overall quality factor (Model 03). These models are compared with hTopoII. For the clarity, we compared few models of our study.

REFERENCES

1. Abramson, J., Adler, J., Dunger, J. et. al., Accurate structure prediction of biomolecular interactions with AlphaFold 3, *Nature*, **630**, 493-500 (2024).
